# Covalent Template-Directed Synthesis: A Powerful Tool for the Construction of Complex Molecules

**DOI:** 10.1021/acs.chemrev.4c00505

**Published:** 2025-01-13

**Authors:** Peter Bolgar, Mohit Dhiman, Diego Núñez-Villanueva, Christopher A. Hunter

**Affiliations:** † Yusuf Hamied Department of Chemistry, University of Cambridge, Lensfield Road, Cambridge CB2 1EW, United Kingdom; ‡ Instituto de Química Médica (IQM-CSIC), C/Juan de la Cierva 3, 28006 Madrid, Spain

## Abstract

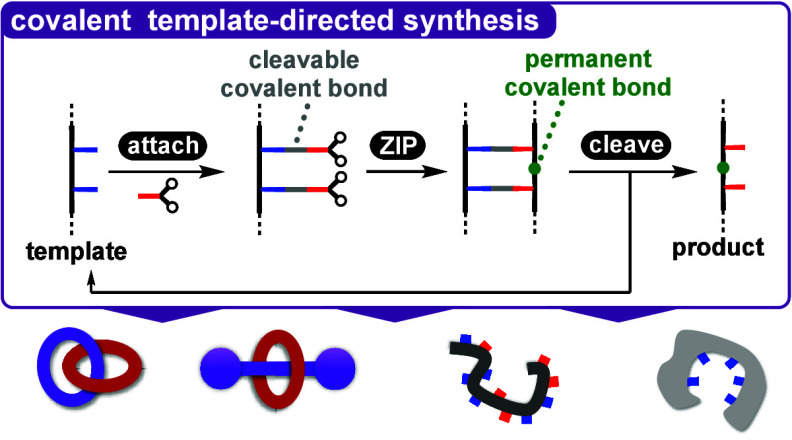

Template-directed synthesis has become a powerful methodology to access complex molecules. Noncovalent templating has been widely used in the last few decades, but less attention has been paid to covalent template-directed synthesis, despite the fact that this methodology was used for the first reported synthesis of a catenane. This review highlights the evolution of covalent templating over the last 60 years, thereby providing a toolbox for the design of efficient covalent templating processes. Covalent templating represents a useful synthetic tool for accessing complex molecules, and the examples described here include the synthesis of macrocycles, mechanically interlocked molecules, linear oligomers, polydisperse linear polymers, and cross-linked polymer networks.

## 1 Introduction

Template-directed synthesis is the basis of DNA replication and governs the transmission of biological inheritance and the expression and regulation of biological function in nature.^1,2^ Soon after the discovery of the structure of DNA and the mechanism of DNA replication, Todd envisaged that chemists would emulate nature and use the principles of template-directed synthesis in organic chemistry.^3^ Template-directed synthesis has since become a versatile tool to synthesize otherwise inaccessible targets.^4−9^

**Figure 1:**
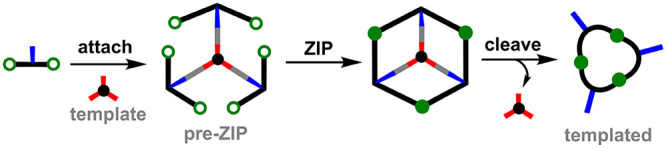
Covalent template-directed synthesis of a macrocycle. The red and blue bars represent complementary groups on the template and reactant, which can be covalently linked in the **attach** step to give the pre-**ZIP** intermediate. The intramolecular **ZIP** reaction connects all of the reactive sites on the reactants (green circles) to give a macrocycle. In the **cleave** step, the gray bonds between the blue and red groups are broken again to release the product from the template.

**Figure 2:**
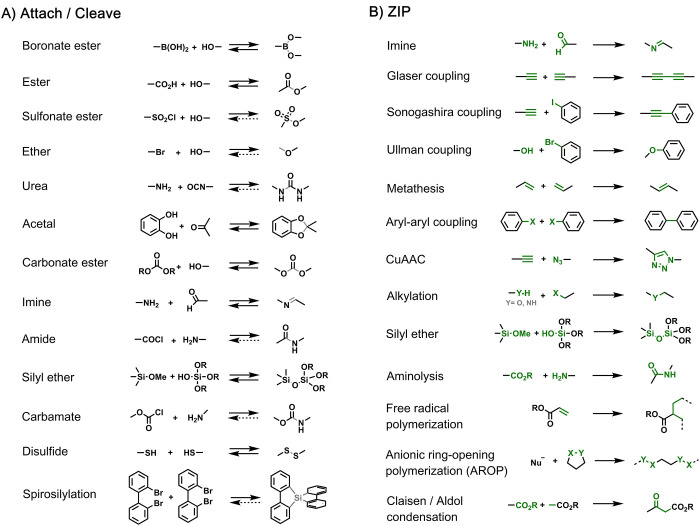
Different kinds of chemistry are compatible with the **attach**/**cleave** and **ZIP** steps. Dashed lines represent the **cleave** reactions which yield different functional groups than those installed in the starting materials.

**Figure 3:**
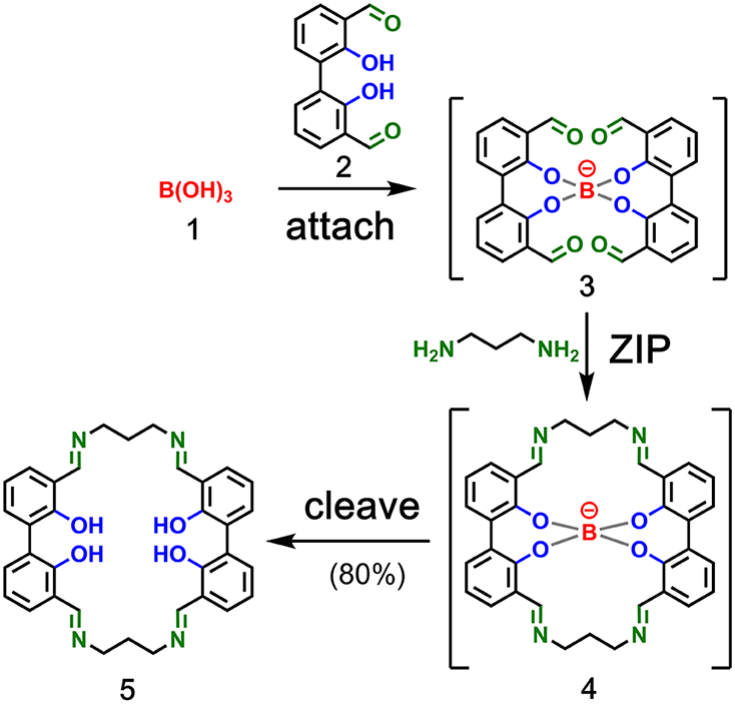
Covalent template-directed synthesis of a macrocycle using boronic ester chemistry for the **attach**/**cleave** steps and imine chemistry for the **ZIP** step. Adapted with permission from ref (33). Copyright 1985 Royal Society of Chemistry.

**Figure 4:**
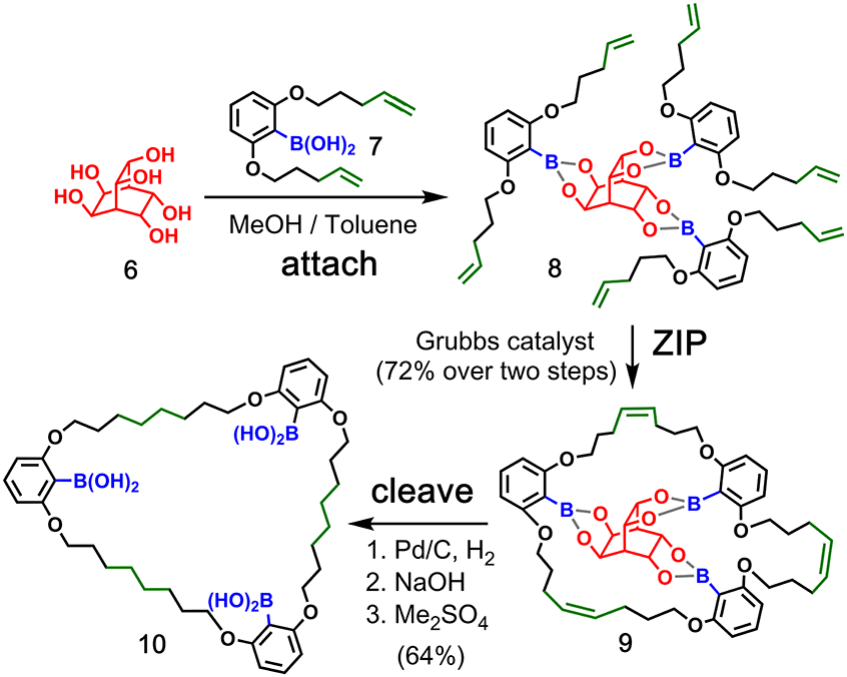
Covalent template-directed synthesis of a trimeric macrocycle using boronic ester chemistry for the **attach**/**cleave** steps and ring-closing metathesis for the **ZIP** step. Adapted with permission from ref (37). Copyright 2013 Royal Society of Chemistry.

In organic synthesis, a template can be defined as a molecular entity able to organize an assembly of atoms in a specific spatial arrangement prior to bond formation. Template-directed synthesis is used to direct formation of a specific product in cases where the reactants have the potential to follow alternative reaction pathways in the absence of template.^10^ Although template effects are often invoked in other contexts, a true template is something that can be removed after the reaction, separated from the product, and reused. Thus, reversible chemistry is required for the attachment of reactants to the template, and this chemistry must be orthogonal to the reaction used for the formation of the templated product. One approach to achieve the necessary reversibility in the attachment of reactants to a template is to employ noncovalent interactions. Noncovalent template-directed synthesis has, therefore, become a well-established field within supramolecular chemistry with numerous examples of the use of metal–ligand coordination, aromatic stacking, salt bridges, and H-bonding to direct the synthesis of products that are usually macrocyclic or mechanically interlocked molecules.^4−15^ The supramolecular approach is actually predated by an alternative strategy based on cleavable covalent bonds, which was first described more than 50 years ago.^16^ Although covalent template-directed synthesis has not been used as much as the noncovalent methodologies, there are important differences that provide advantages for the synthesis of some classes of target molecule. This review highlights the value of covalent templating as a complementary tool to noncovalent approaches for the synthesis of complex molecular architectures. Criteria for the design of efficient covalent templating systems together with a survey of successful applications of covalent template-directed synthesis over the last five decades provide a perspective on where future opportunities may lie in this field.

**Table 1:**
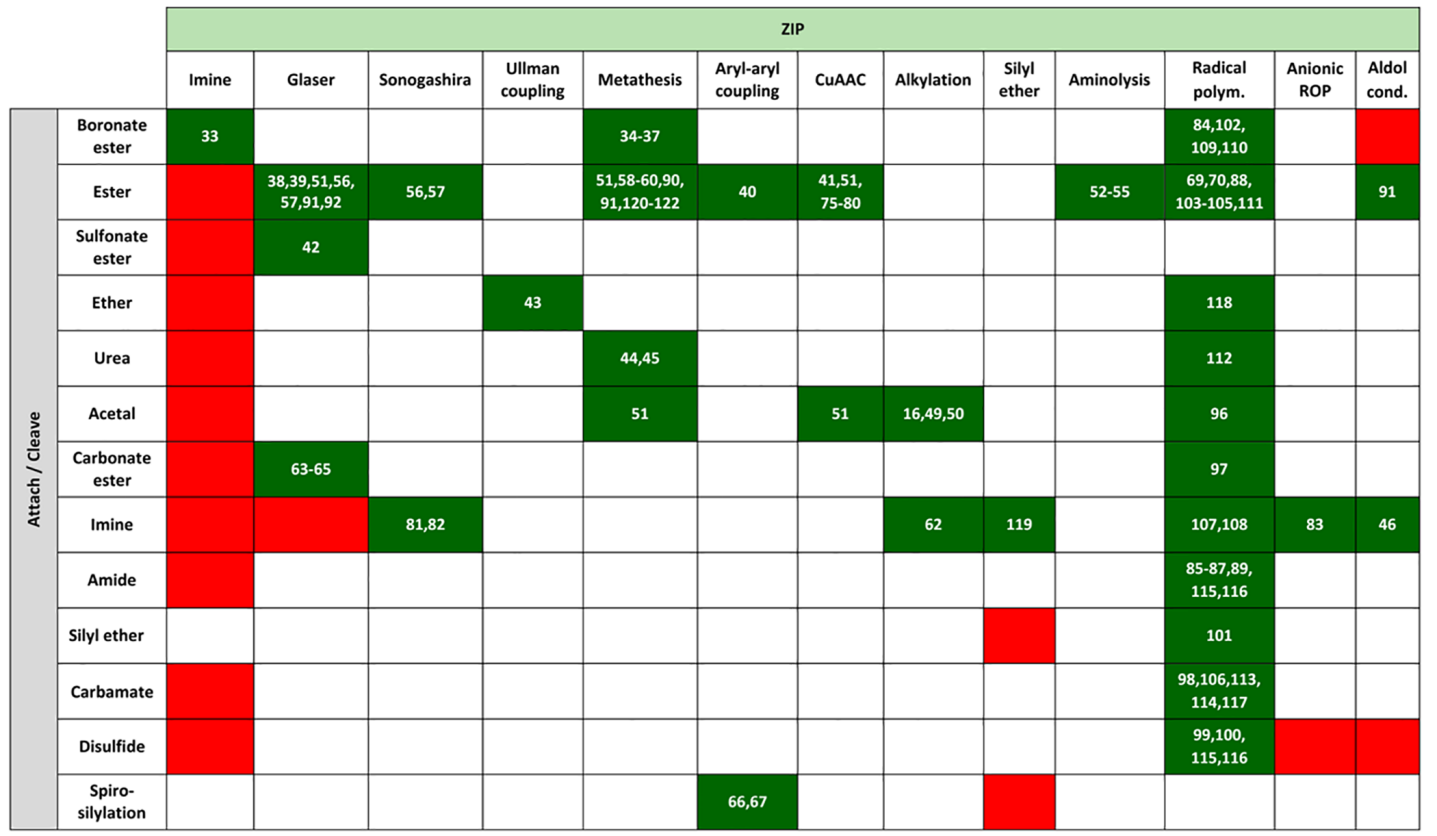
Compatibility Matrix for Chemistry Used in the **Attach**/**Cleave** and **ZIP** Steps in Covalent Template-Directed Synthesis^a^

a Green: reported (references listed); Red: not reported and likely incompatible reactions; Blank: not reported and likely compatible reactions. ROP: ring opening polymerization.

**Figure 5:**
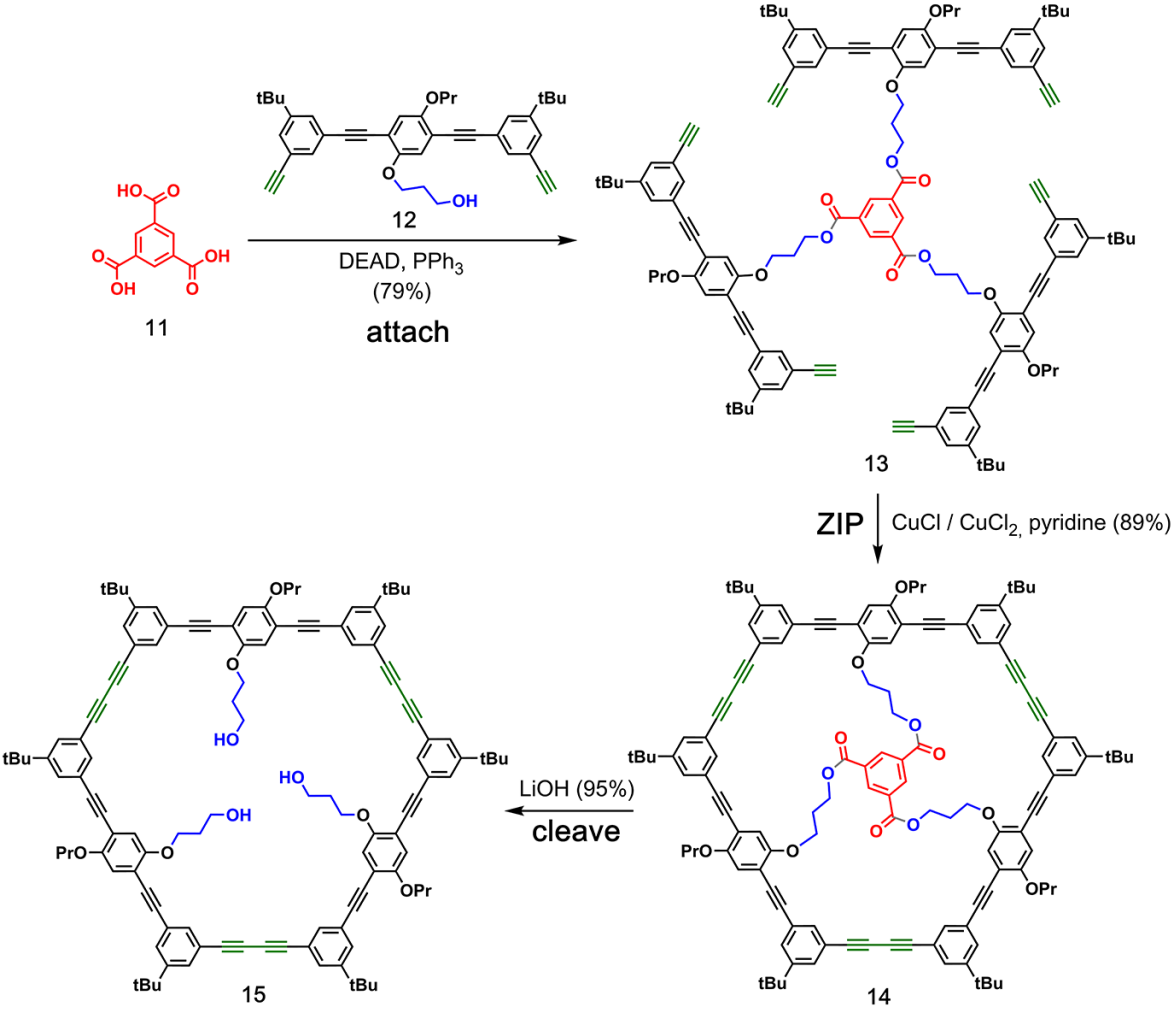
Covalent template-directed synthesis of a macrocycle using ester chemistry for the **attach**/**cleave** steps and Glaser coupling for the **ZIP** step. Adapted with permission from ref (38). Copyright 1997 American Chemical Society.

**Figure 6:**
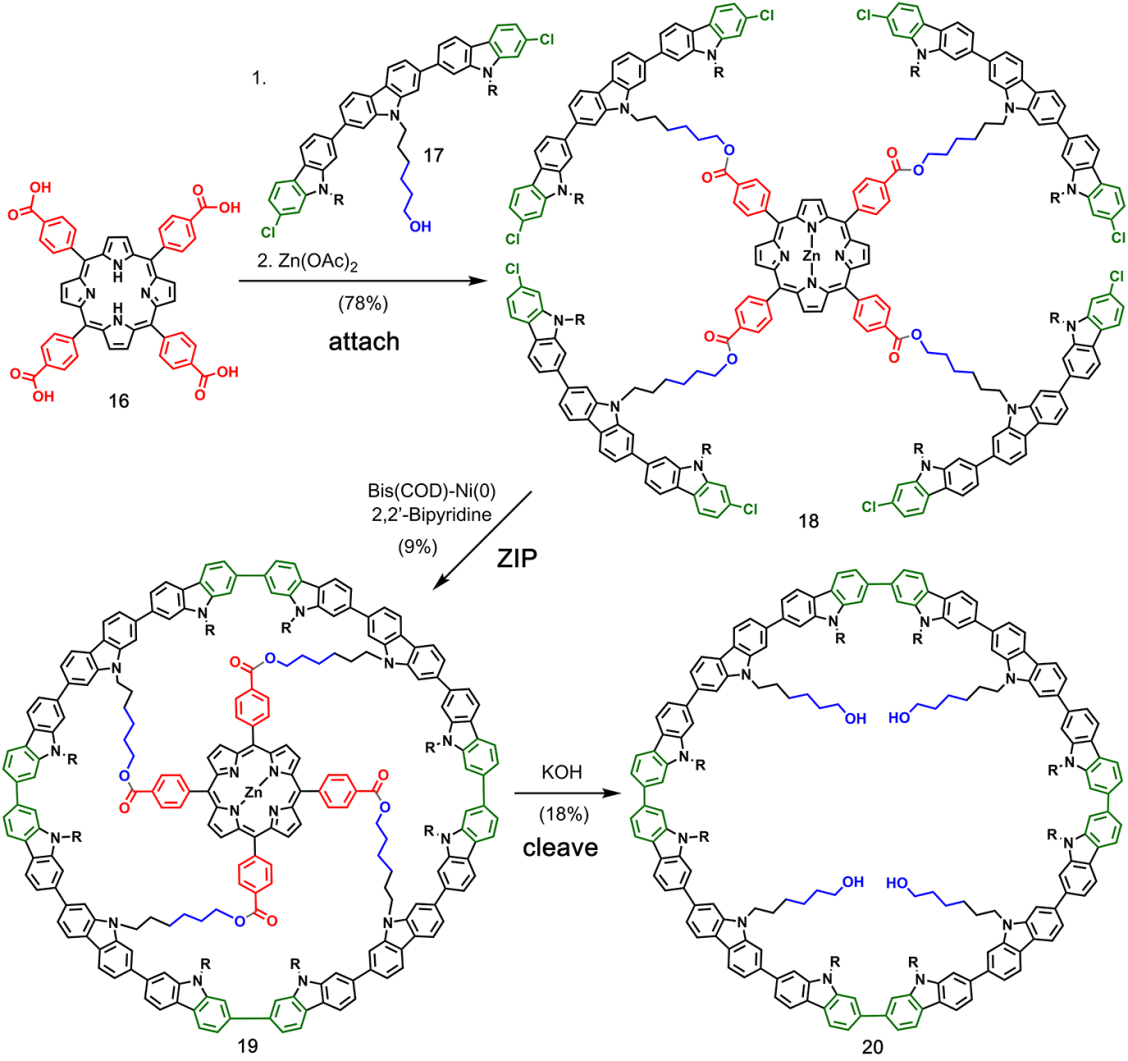
Covalent template-directed synthesis of a conjugated polycarbazole macrocycle using a porphyrin as a template for cyclization via Yamamoto coupling; R: 2-hexyldecyl. Adapted with permission from ref (40). Copyright 2006 Wiley.

**Figure 7:**
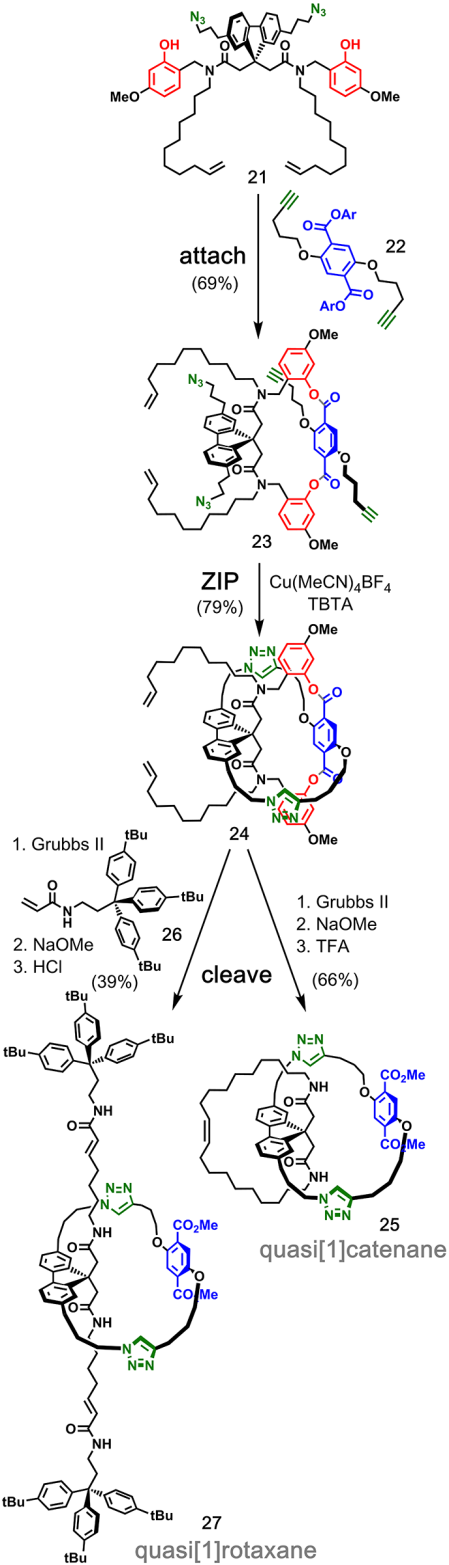
Covalent template-directed synthesis of a macrocycle as a starting point for topologically complex molecules. Adapted with permission from ref (41). Copyright 2017 Springer Nature.

An early classification of template-directed methods focused on the difference between kinetic and thermodynamic templates regarding the nature of the reaction involved in the formation of the product: thermodynamic templates operate by shifting the equilibrium of a reversible reaction by preferential stabilization of one of the components; kinetic templates operate in irreversible reactions by stabilizing a key transition state.^17,18^ Here, we focus on the nature of the interaction involved in the attachment of the reactants to the template. Figure 1 illustrates the key steps and intermediates involved in covalent template-directed synthesis and introduces the terminology and color coding that will be used throughout this review. First, a kinetically stable covalent bond is used to **attach** functional groups on the reactants (blue) to functional groups on the template (red). A high-yielding and efficient reaction should be selected to ensure the quantitative assembly of this pre-**ZIP** intermediate. In the **ZIP** step, an intramolecular reaction is used to couple functional groups on the reactants (green) to obtain the product that is covalently attached to the template. Alternative intermolecular reaction pathways will not compete with the intramolecular templated pathway if the concentration of the pre-**ZIP** intermediate is significantly lower than the effective molarity for the intramolecular reactions (EM). A third reaction is then used to **cleave** the gray bonds that attach the product to the template. Although three synthetic steps are required for the complete templating process in Figure 1, the covalent methodology offers a number of advantages over the noncovalent approach:

1. Complete loading of reactants onto the template can be guaranteed in a covalent process, whereas the pre-**ZIP** intermediate is an equilibrium with partially loaded templates in a noncovalent process.
2. In a covalent process, competing intermolecular reaction pathways can be minimized by using very dilute conditions that would lead to dissociation of the reactants from the template in a noncovalent process.
3. The robustness of the covalent bonds that attach the reactants to the template means that a much wider range of conditions can be tolerated for the **ZIP** reaction compared with noncovalent templating, which is not compatible with harsh conditions.
4. In a covalent process, the **attach** and **ZIP** processes are two separate steps so that the covalent intermediates can be isolated and characterized, allowing each step to be optimized independently.
5. Cleavage of products covalently attached to a template is an irreversible process, facilitating separation.

**Figure 8:**
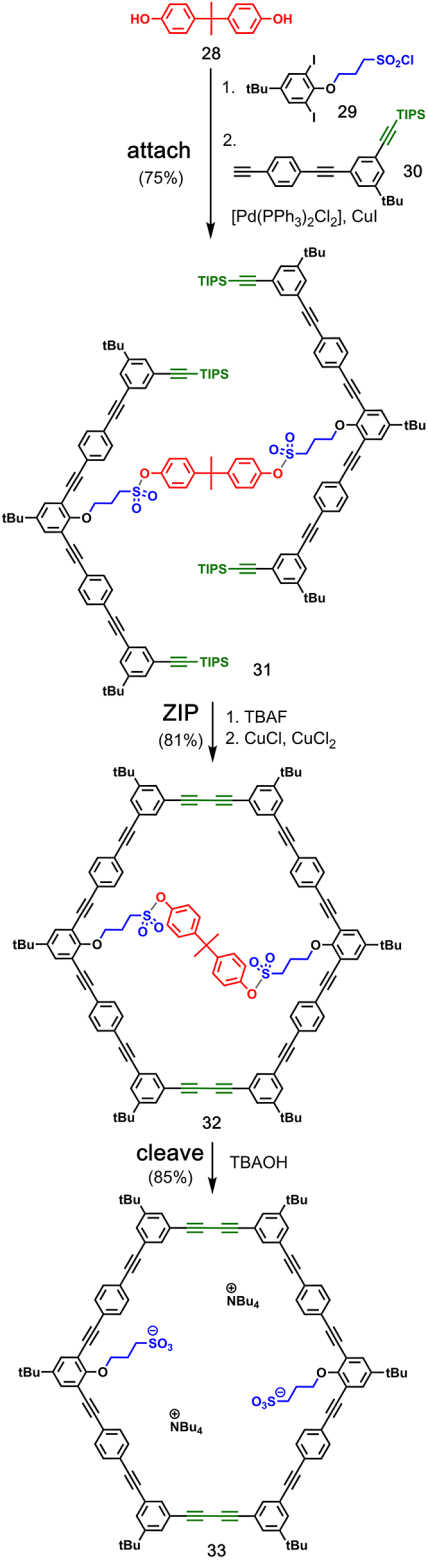
Covalent template-directed synthesis of a macrocycle using sulfonate ester chemistry in the **attach**/**cleave** steps and Glaser coupling for the **ZIP** step. Adapted with permission from ref (42). Copyright 2003 Wiley.

**Figure 9:**
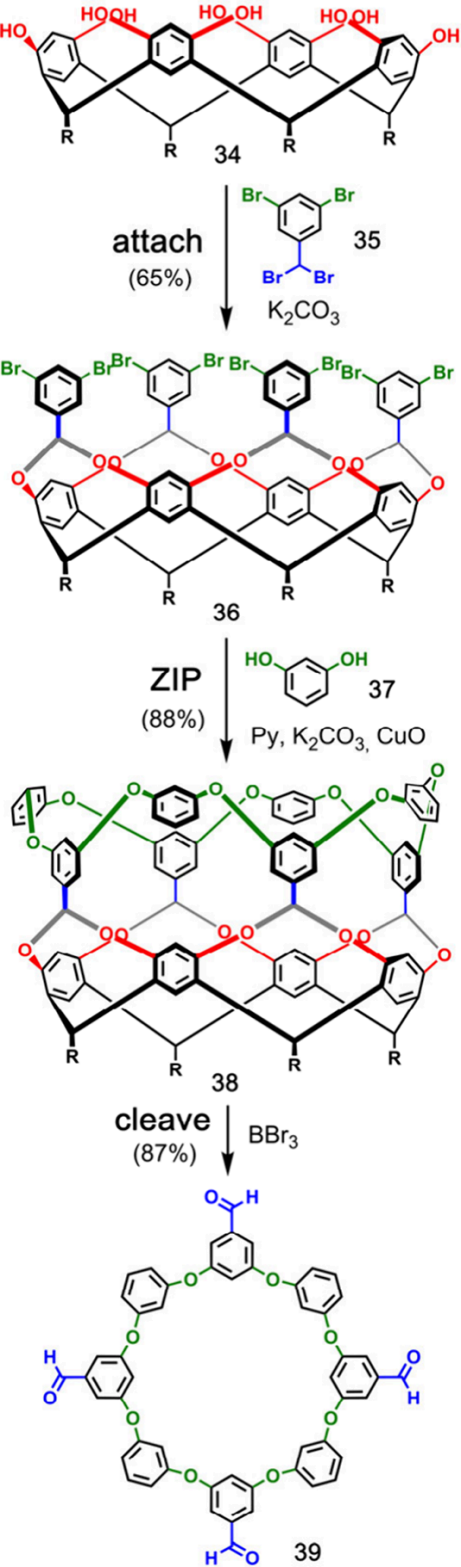
Covalent template-directed synthesis of an aromatic crown ether macrocycle using a resorcinarene as a template for cyclization via Ullman ether reactions; R: −CH_2_CH_2_Ph. Adapted with permission from ref (43). Copyright 2003 American Chemical Society.

Successful implementation of the approach outlined in Figure 1 requires orthogonal chemistry for the bonds used in the **attach**/**cleave** steps and the bonds formed in the **ZIP** step. Reactions from the protecting group chemistry toolbox are particularly interesting in this context because they have been specifically optimized for high-yielding attachment and cleavage reactions that are compatible with a wide range of chemical functionality. Figure 2 shows different kinds of reactions that could be used in the **attach**/**cleave** and **ZIP** steps, and their compatibility is analyzed in Table 1. Examples of reaction combinations that have been successfully utilized in covalent template-directed synthesis are highlighted in the green boxes. For the **attach** step, ester coupling is clearly one of the most useful reactions. Ester formation is high-yielding, and the products are easy to purify, especially when using water-soluble coupling reagents. Moreover, ester hydrolysis, which is required for the **cleave** step, proceeds under mild conditions that are compatible with most functional groups. For the **ZIP** step, there are many examples of the use of free radical polymerization for template-directed synthesis of polymeric materials. Metal-catalyzed coupling reactions, such as Glaser and Sonogashira, olefin metathesis, and copper(I)-catalyzed alkyne–azide cycloaddition (CuAAC) are all high-yielding reactions that are compatible with a wide range of functional groups. These reactions have all been successfully implemented in the **ZIP** step for the covalent template-directed synthesis of discrete small molecules. Combinations of coupling reactions that are not orthogonal and are likely to prove problematic for applications in covalent templating are highlighted as red boxes in Table 1. However, there are a large number of empty boxes in Table 1, and these combinations correspond to approaches that have not been used in covalent template-directed syntheses but match the orthogonality criteria. For example, acetal and carbamate functional groups are commonly used in protecting group chemistry and are compatible with high-yielding coupling reactions, such as Glaser or CuAAC.^19−22^ The analysis in Table 1 provides a starting point for the design of new synthetic methods based on covalent templating but includes only reactions that have been reported for this purpose. Additional types of reaction can be imagined: for example, the **ZIP** step could make use of any of the recently developed “click” chemistries, including Diels–Alder reactions, thiol–ene coupling, strain-promoted alkyne–azide cycloadditions, photoclick reactions, as well as ring-opening oligomerization of strained heterocycles.^23,24^

In organic synthesis, tethering has been developed as an effective strategy for installing functionalities that would otherwise be challenging due to limitations in the regio- or stereoselectivity of the reaction.^25−27^ Tethering and covalent templating are closely interlinked concepts as both exploit intramolecular reactions to achieve selectivity. Formally, a tether can be considered a covalent template for the formation of a single covalent bond. There have been several comprehensive reviews of tethering in organic synthesis, so this aspect of covalent templating will not be discussed in detail here.^25−31^

**Figure 10:**
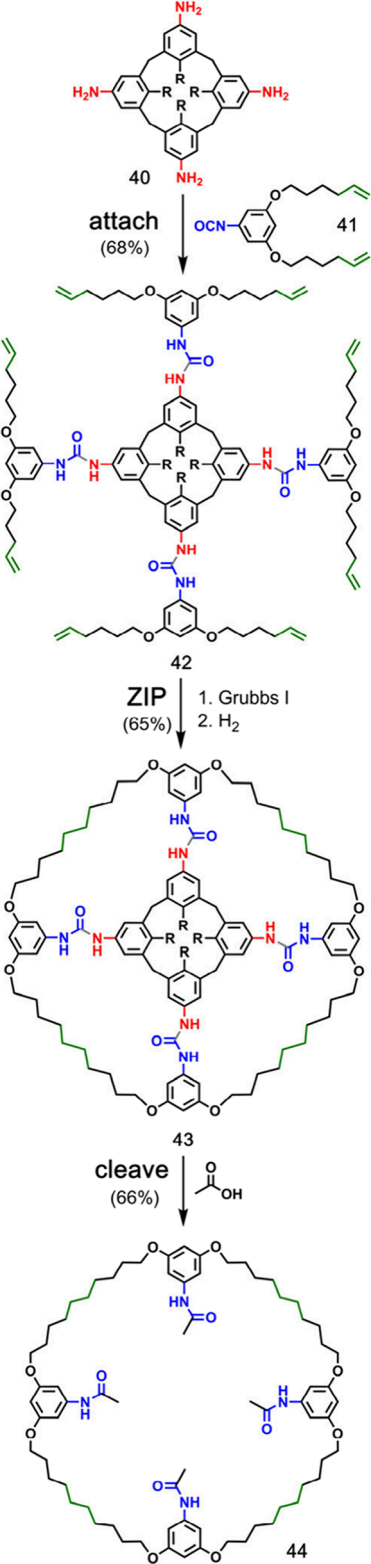
Covalent template-directed synthesis of a macrocycle using a calixarene as a template for cyclization via ring-closing metathesis; R: O–C_5_H_11_. Adapted with permission from ref (44). Copyright 2005 Royal Society of Chemistry.

**Figure 11:**
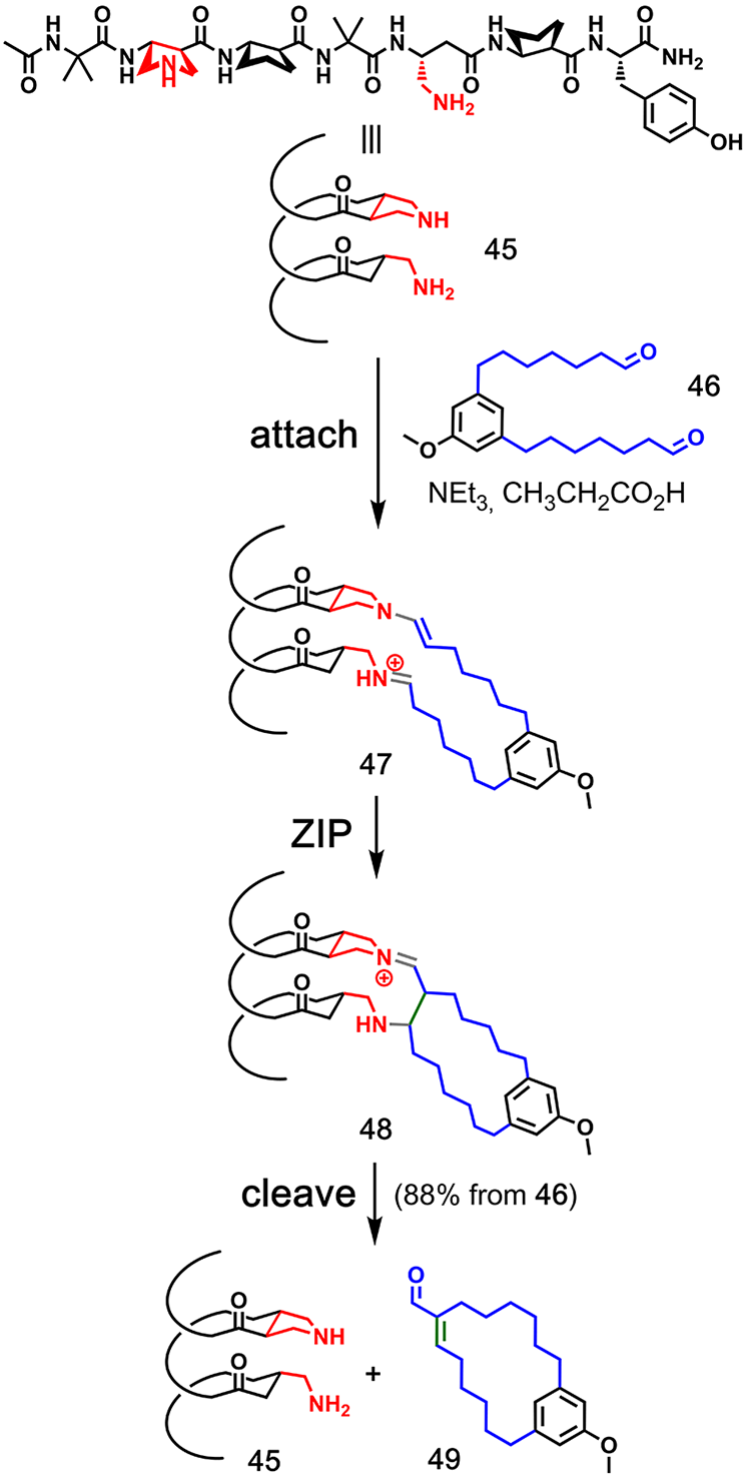
Catalytic covalent template-directed synthesis of a 16-membered macrocycle via an aldol reaction. The foldamer template adopts a helical conformation that presents the reactive primary and secondary amine sites in the correct position for efficient macrocyclization. Adapted with permission from ref (46). Copyright 2019 The Authors.

## 2 Macrocycles

The synthesis of macrocycles has been an interest of organic chemists for more than a century. Macrocycles usually display distinct properties in comparison with those of acyclic analogues. A number of interesting applications have been developed in supramolecular chemistry, and macrocyclic receptors for organic and inorganic guest molecules have been exploited as sensors, tools for organic synthesis, and traps for pollutants. Template-directed synthesis is widely used for the synthesis of macrocycles because the template can favor the preferential formation of a specific macrocycle over the statistical mixture of different macrocycles and linear polymers that would typically be obtained in an oligomerization reaction.^32^

**Figure 12:**
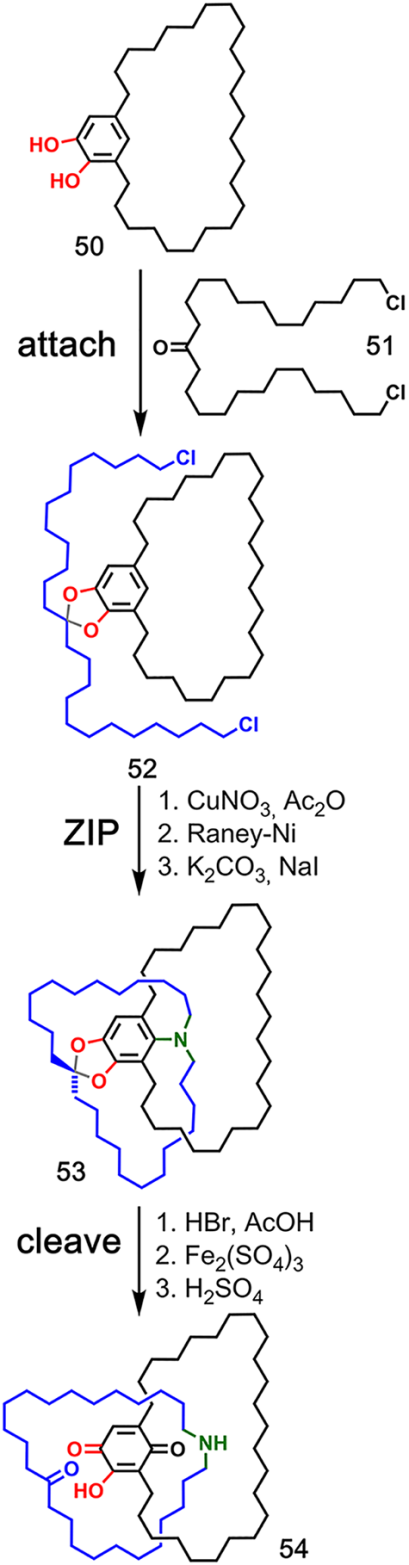
Covalent bond-directed synthesis of a [2]catenane. Adapted with permission from ref (16). Copyright 1964 Wiley.

**Figure 13:**
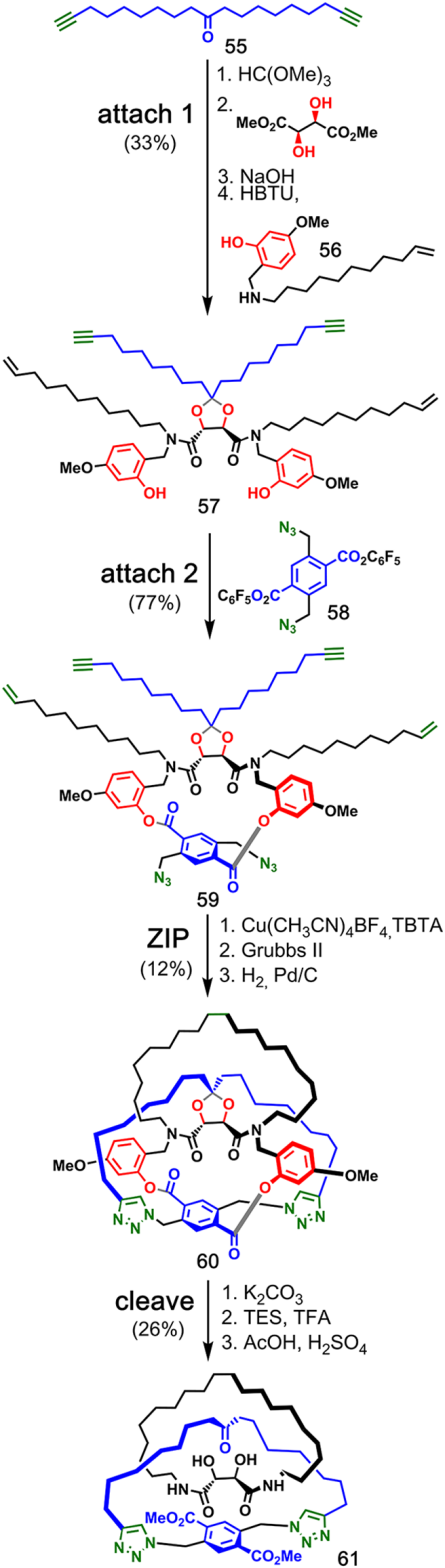
Covalent bond-directed synthesis of a [2]catenane using CuAAC and metathesis reactions to close the rings prior to the hydrolysis of the ketal. Adapted with permission from ref (51). Copyright 2021 Wiley.

### 2.1 Boronate Ester Covalent Attachment

The first covalent template-directed synthesis of a simple macrocycle was reported in 1985 by Moneta et al. (Figure 3).^33^ In this example, boronic acid (**1**, red) acted as a covalent template by bringing together two molecules of a bis-phenol **2** through formation of a boronate adduct (**3**). Each bis-phenol carried two aldehyde groups (green), which were connected as imines in the **ZIP** step to give **4** before the boron–oxygen bonds were simultaneously cleaved to release the macrocyclic product **5** in 80% yield.

Boronic acid chemistry was also exploited by Lüning and co-workers for the covalent template-directed synthesis of a series of macrocycles using bicyclic and tetracyclic tetrols and hexols as templates.^34−37^Figure 4 shows the templated synthesis of a trimeric macrocycle **10**.^37^ The **attach** step involves boronic ester formation between hexol template **6** and boronic acid monomer **7**. The resulting triboronate **8** was cyclized in the **ZIP** step by ring-closing metathesis to yield the trimeric macrocycle **9** as a diastereomeric *E*/*Z* mixture. Catalytic hydrogenation yielded a single saturated macrocycle, and the **cleave** step hydrolyzed the boronic esters to regenerate the template and produce the macrocycle **10**.

**Figure 14:**
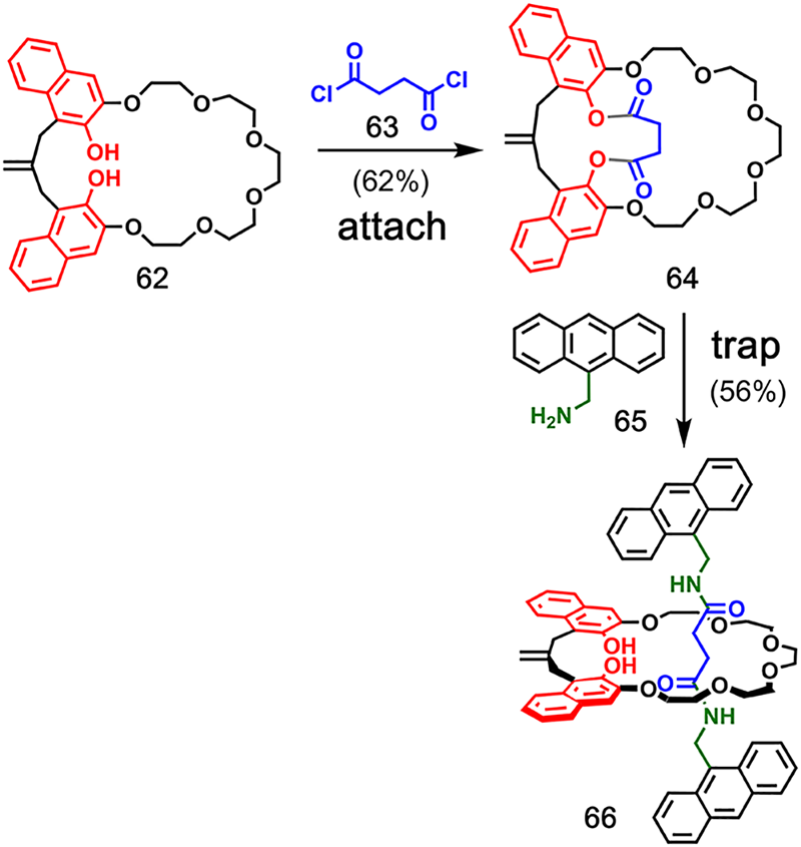
Covalent bond-directed synthesis of a [2]rotaxane utilizing ester aminolysis. Adapted with permission from ref (52). Copyright 2002 Elsevier.

**Figure 15:**
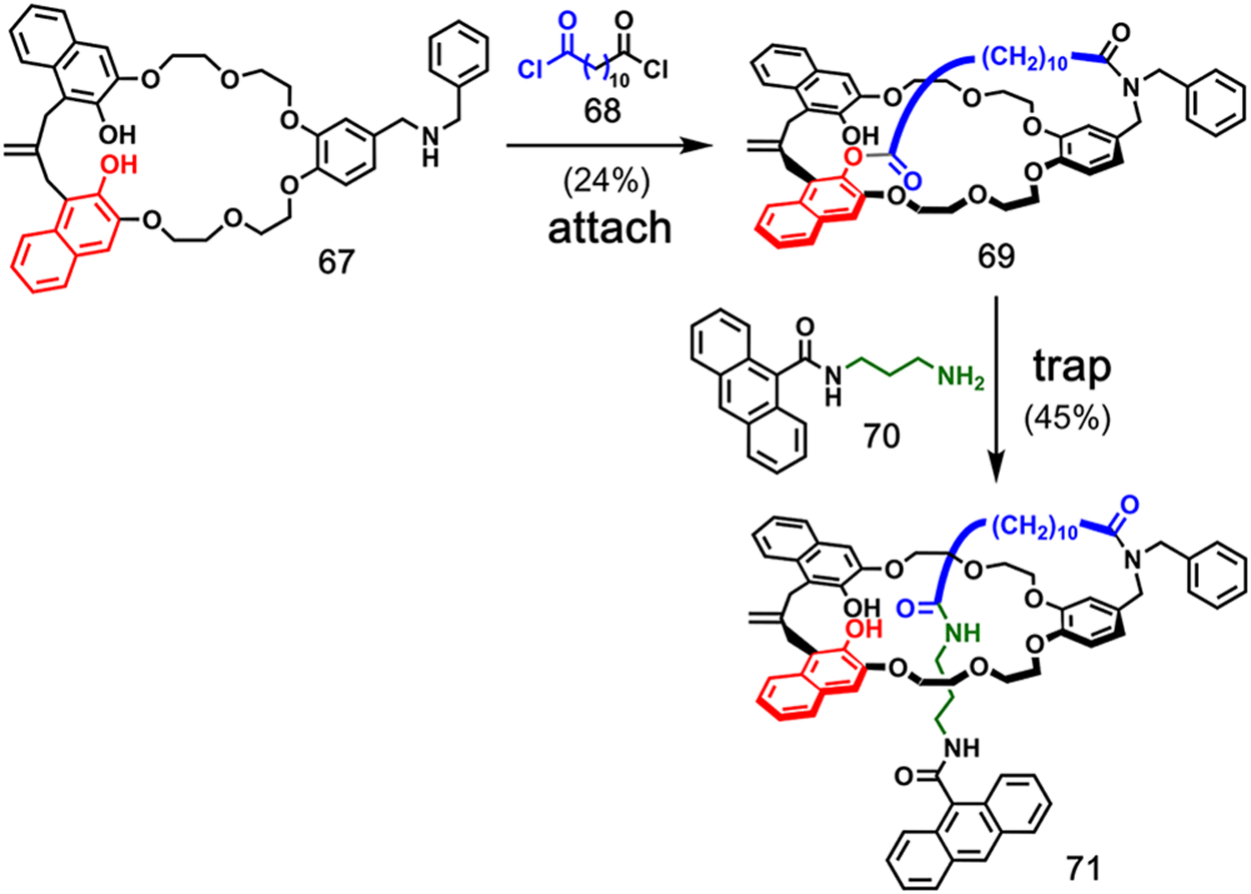
Covalent bond-directed synthesis of a [1]rotaxane utilizing ester aminolysis. Adapted with permission from ref (53). Copyright 2004 American Chemical Society.

**Figure 16:**
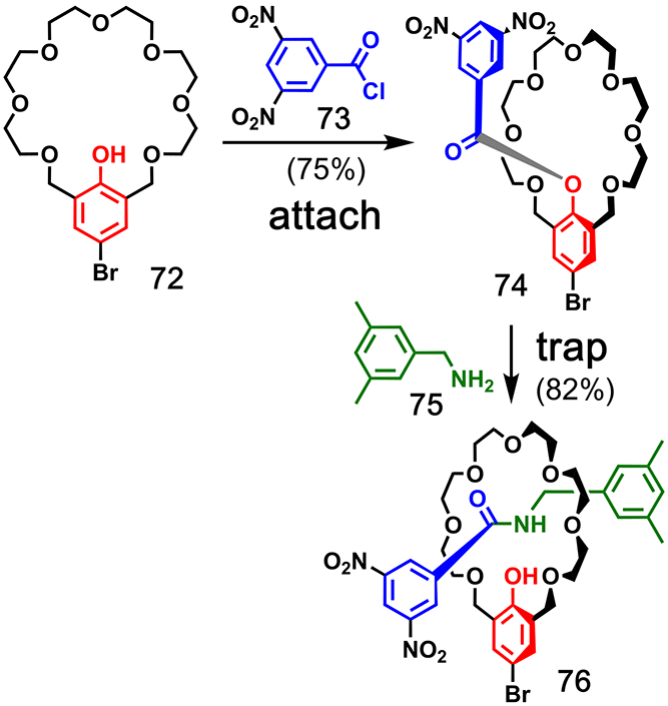
Covalent bond-directed synthesis of a [2]rotaxane utilizing ester aminolysis. Adapted with permission from ref (55). Copyright 2007 American Chemical Society.

### 2.2 Ester Covalent Attachment

Ester chemistry has been widely used in covalent template-directed macrocyclization reactions. Höger and co-workers used covalent templating to make larger macrocycles by using ester chemistry for covalent attachment and cleavage and Glaser coupling for the **ZIP** reaction. Figure 5 shows how three building blocks bearing terminal alkyne groups (**12**) were attached to a trimesic acid template (**11**).^38^ The triester **13** was then subjected to Glaser coupling to yield a 54-membered macrocycle (**14**) in 89% yield. In the absence of the template, a mixture of cyclic oligomers and noncyclic polymers was formed with only 20% yield of the cyclic trimer. The final **cleave** step was achieved by base-catalyzed hydrolysis of **14**, which provided the macrocycle **15** in excellent yield. A subsequent work by Höger and co-workers also described a variation on this approach where two dialkyne building blocks were assembled on the template before macrocyclization. This methodology was used in the synthesis of dimeric heteromacrocycles.^39^

Müllen and co-workers reported the templated synthesis of a fully conjugated polycarbazole macrocycle (Figure 6).^40^ Tris(carbazole) building block **17** was attached to tetraphenylporphyrin template **16** via ester linkages before complexation with zinc afforded the pre-**ZIP** intermediate **18**. Yamamoto coupling of adjacent carbazole rings gave the cyclized product **19**, and then, the template was removed by hydrolysis of the esters. The target macrocycle **20** was isolated in just 1% overall yield.

Ester-based covalent templating has been also applied for the syntheses of a quasi[1]catenane and a quasi[1]rotaxane, both with a spiro geometry arising from a tetrahedral carbon center (Figure 7).^41^ Starting from a precursor equipped with phenol, azide, and alkene functional groups (**21**), the aryl dialkyne **22** was attached via transesterification giving macrocyclic intermediate **23**. The second macrocyclic ring was then closed using CuAAC to provide **24**. In one pathway, subsequent ring-closing metathesis gave the final backfolding ring closure, and the template was removed by transesterification and protolytic cleavage to afford quasi[1]catenane **25** in 36% overall yield. In the other pathway, cross-metathesis was used to stopper the alkenes with acrylamide derivative **26**, and transesterification and protolysis removed the template to afford the quasi[1]rotaxane **27** in 21% overall yield.

**Figure 17:**
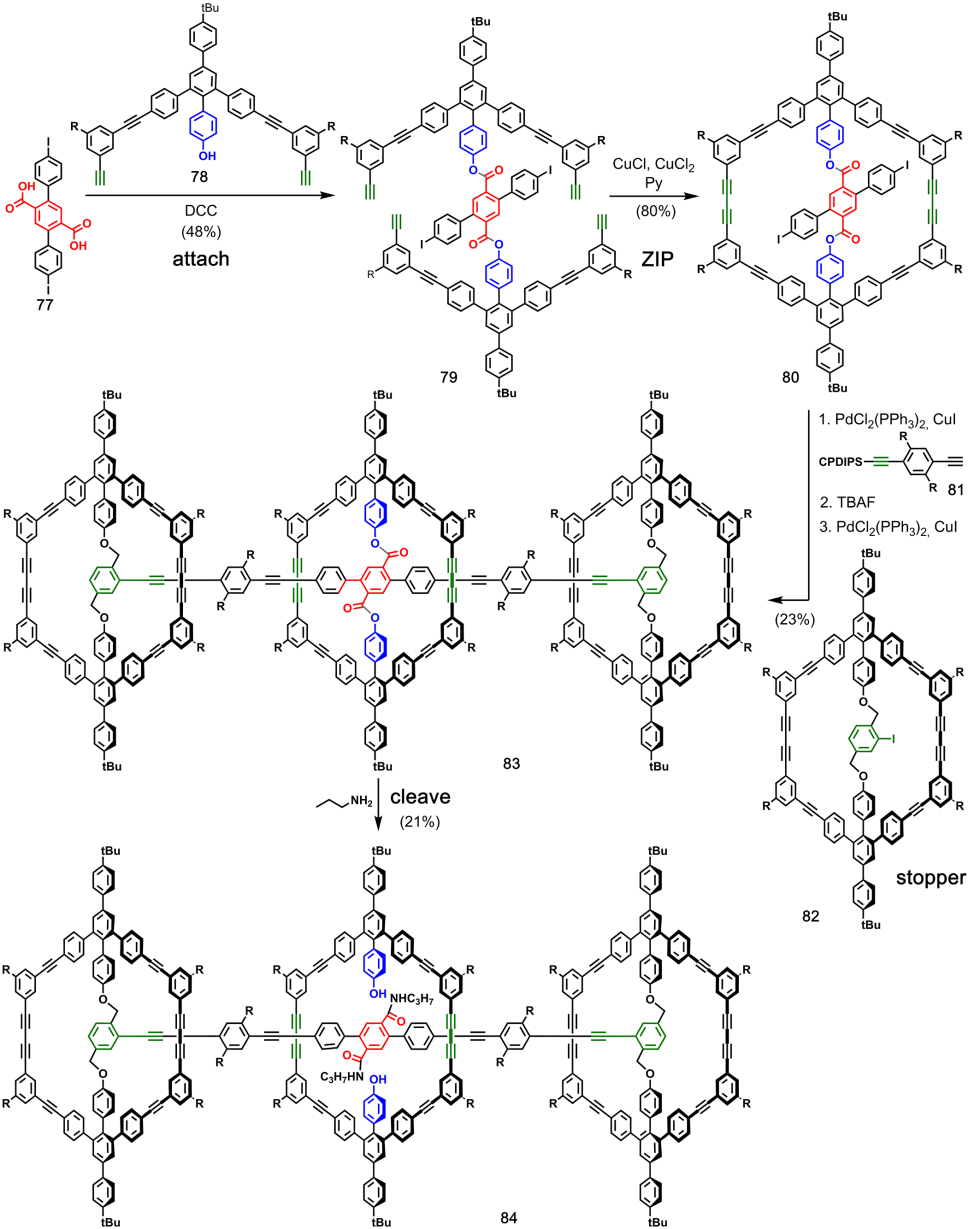
Covalent bond-directed synthesis of a [2]rotaxane using ester chemistry in the **attach**/**cleave** steps and Glaser and Sonogashira couplings in the **ZIP** step; R: O–C_16_H_33_. Adapted with permission from ref (56). Copyright 2016 Wiley.

### 2.3 Miscellaneous

Höger and co-workers also reported the use of sulfonate esters for the **attach**/**cleave** chemistry.^42^ Bis-phenol template **28** was loaded with sulfonyl chloride **29** followed by Sonogashira coupling with alkyne **30** to provide pre-**ZIP** intermediate **31** (Figure 8). After alkyne deprotection, the **ZIP** step was successfully carried out by Glaser coupling to give compound **32**. Finally, the aryl sulfonate esters in **32** were cleaved by reaction with tetrabutylammonium hydroxide to give tetrabutylammonium disulfonate macrocycle **33** in 85% yield.

**Figure 18:**
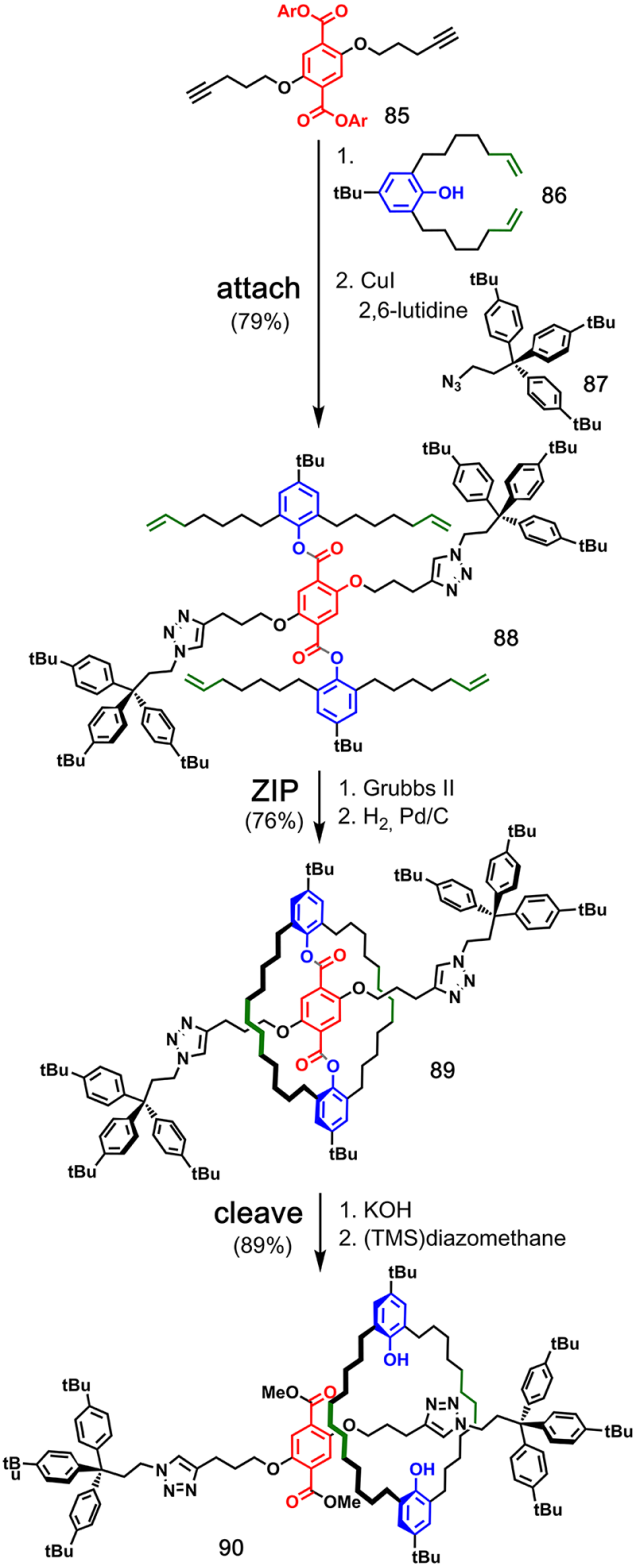
Covalent bond-directed synthesis of a [2]rotaxane using ester chemistry in the **attach**/**cleave** steps and metathesis in the **ZIP** step. Adapted with permission from ref (58). Copyright 2017 American Chemical Society.

**Figure 19:**
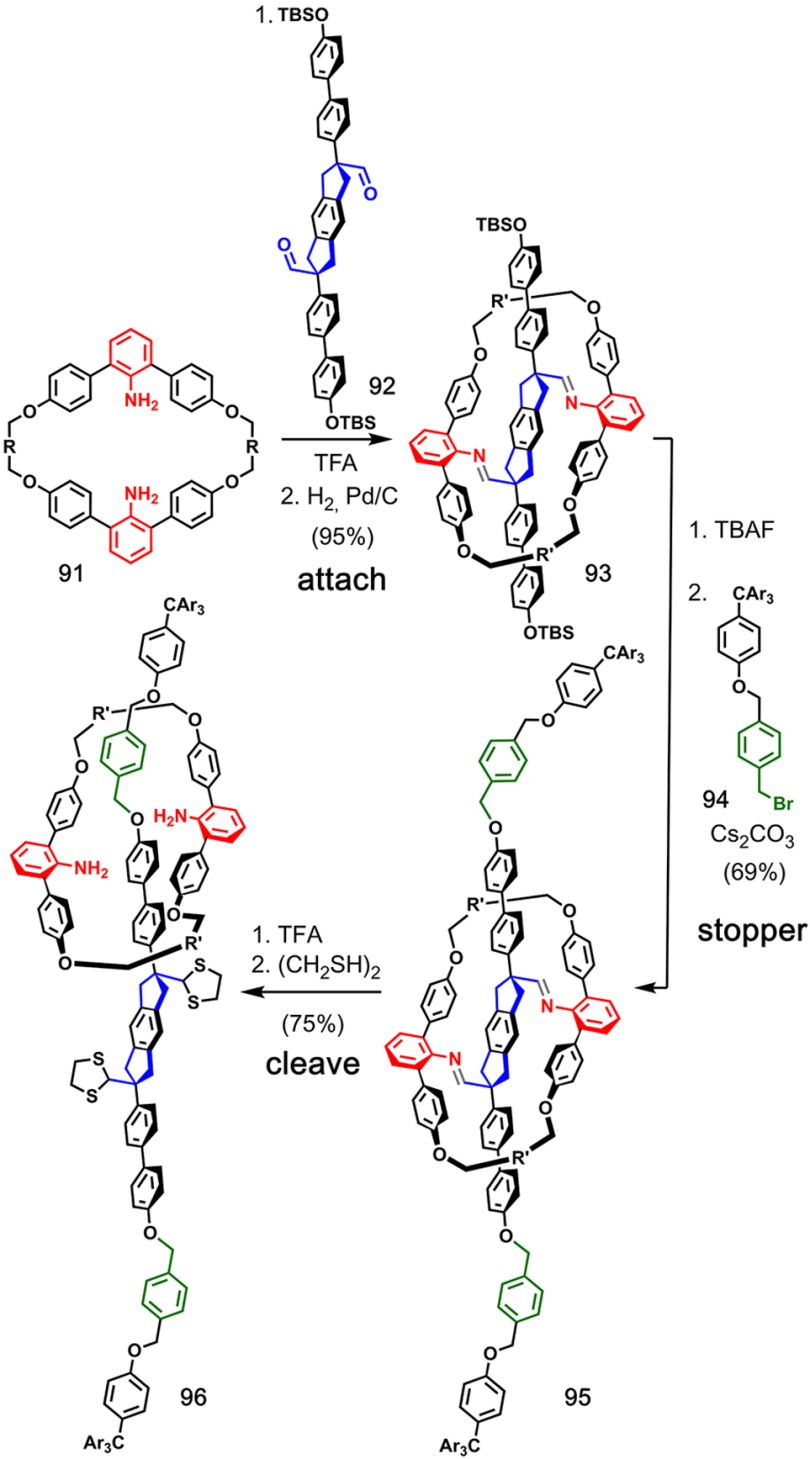
Dynamic covalent-bond-directed [2]rotaxane synthesis using imine chemistry in the **attach**/**cleave** steps; R: – C≡C–C≡C-; R’: -(CH_2_)_4_-; Ar: 4′-*tert*-butylbiphenyl. Adapted with permission from ref (62). Copyright 2006 Wiley.

Figure 9 shows the templated synthesis of aromatic crown ether **39** reported by Gibb and co-workers.^43^ The resorcinarene template **34** was bridged with the benzal bromide **35**. The **ZIP** reaction of the resulting product **36** involved eight Ullman ether reactions with resorcinol (**37**), and then, the resorcinarene template was removed from **38** by treatment with BBr_3_ to give the macrocyclic product **39** in 50% overall yield.

Calixarenes have also been used in the templated macrocycle synthesis. In an example by Böhmer and co-workers, four aryl isocyanates bearing terminal alkenes (**41**) were covalently attached to calixarene **40** via urea linkages (Figure 10).^44^ Ring-closing metathesis connected the alkenes in **42** to give cyclic product **43** before the template was cleaved by refluxing in acetic acid. The macrocycle product **44** was isolated in 66% yield.^45^

**Figure 20:**
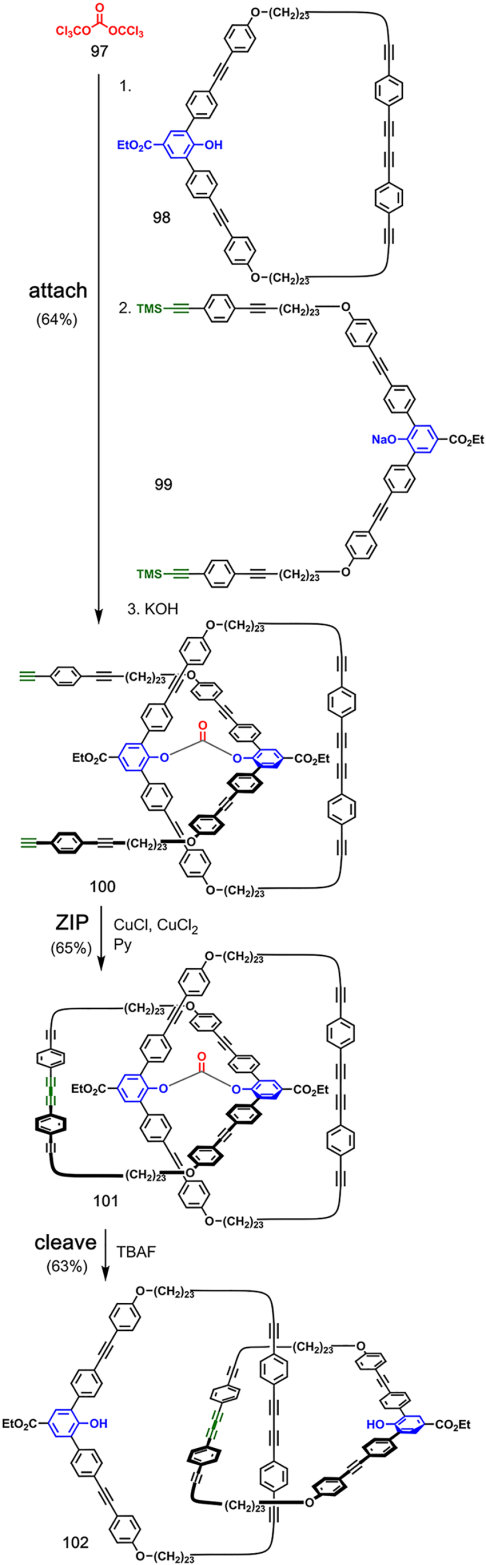
Covalent bond-directed synthesis of a [2]catenane using carbonate ester chemistry in the **attach**/**cleave** steps and Glaser coupling in the **ZIP** step. Adapted with permission from ref (63). Copyright 1999 Wiley.

**Figure 21:**
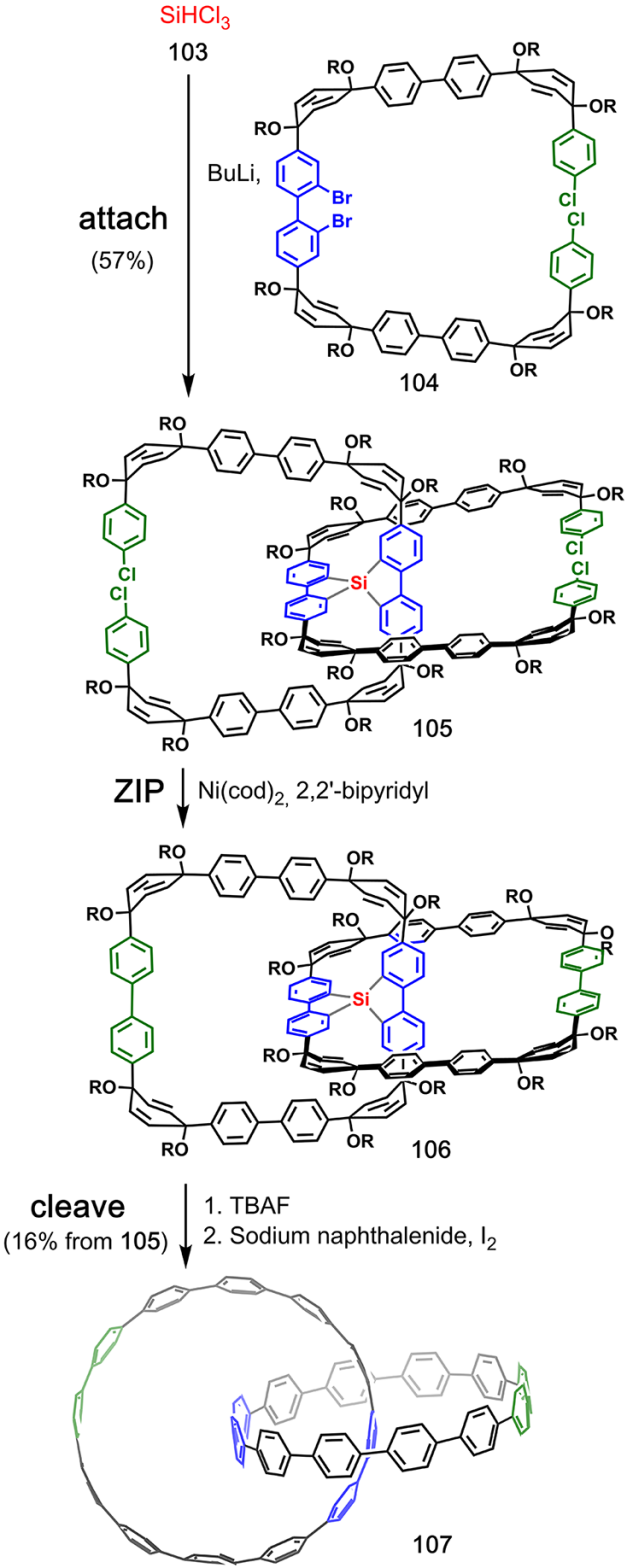
Covalent bond-directed synthesis of an all-benzene catenane using spirosilylation chemistry in the **attach**/**cleave** steps and aryl–aryl coupling in the **ZIP** step; R: *n*-butyl. Adapted with permission from ref (66). Copyright 2019 The Authors.

Figure 11 shows an example of a biologically inspired catalytic covalent template-directed macrocycle synthesis from Gellman and co-workers.^46^ This approach used an α/β-peptide foldamer (**45**) that activates both ends of linear dialdehyde **46** for ring closure via an aldol reaction. It differs from the examples shown previously in that the template was only used in 10 mol %. The template adopts a helical conformation, which contains both a primary and a secondary amine held in a specific arrangement. The proposed mechanism suggests the formation of an enamine and a protonated imine in the **attach** step (**47**), followed by macrocyclization to 16-membered *E*-enal **49** via the formation of intermediate **48**. Efficient templated macrocyclization was also observed for the synthesis of 12-, 14-, 18-, and 22- membered rings.

These examples highlight that ester chemistry is one of the most used methodologies to access macrocycles via covalent templating. The robustness of the ester linkage is a key feature enabling combination with a variety of high-yielding **ZIP** chemistries, such as ring-closing metathesis, Glaser coupling, and CuAAC. Dynamic covalent chemistry involving imines and boronic esters represents a viable alternative, which is open to further development, because there are a number of compatible reactions that have not been explored. For example, there are no examples of the use of hydrazone chemistry for **attach**/**cleave** chemistry in conjunction with CuAAC, Glaser coupling, or Sonogashira coupling for the **ZIP** step.

**Figure 22:**
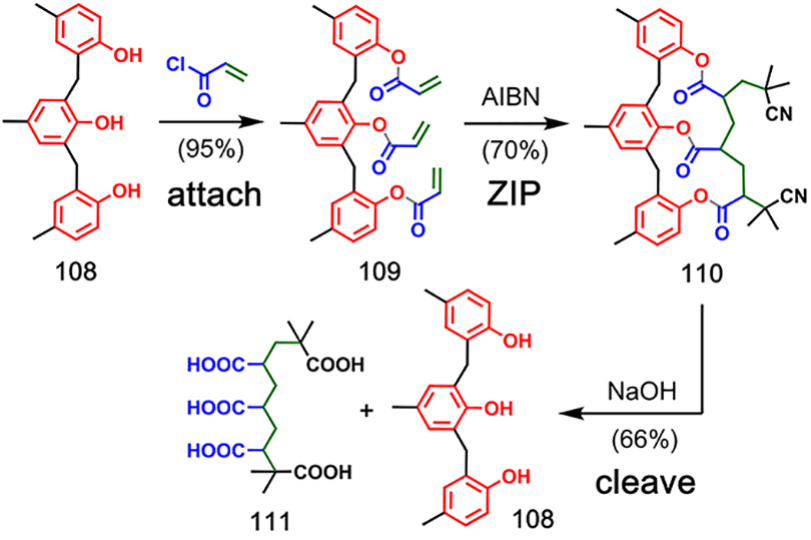
Covalent template-directed synthesis of a pentacarboxylic acid using free radical oligomerization of acrylate monomers attached to a triphenol template using ester chemistry. Adapted with permission from ref (69) and ref (70). Copyright 1966 and 1967 Wiley.

**Figure 23:**
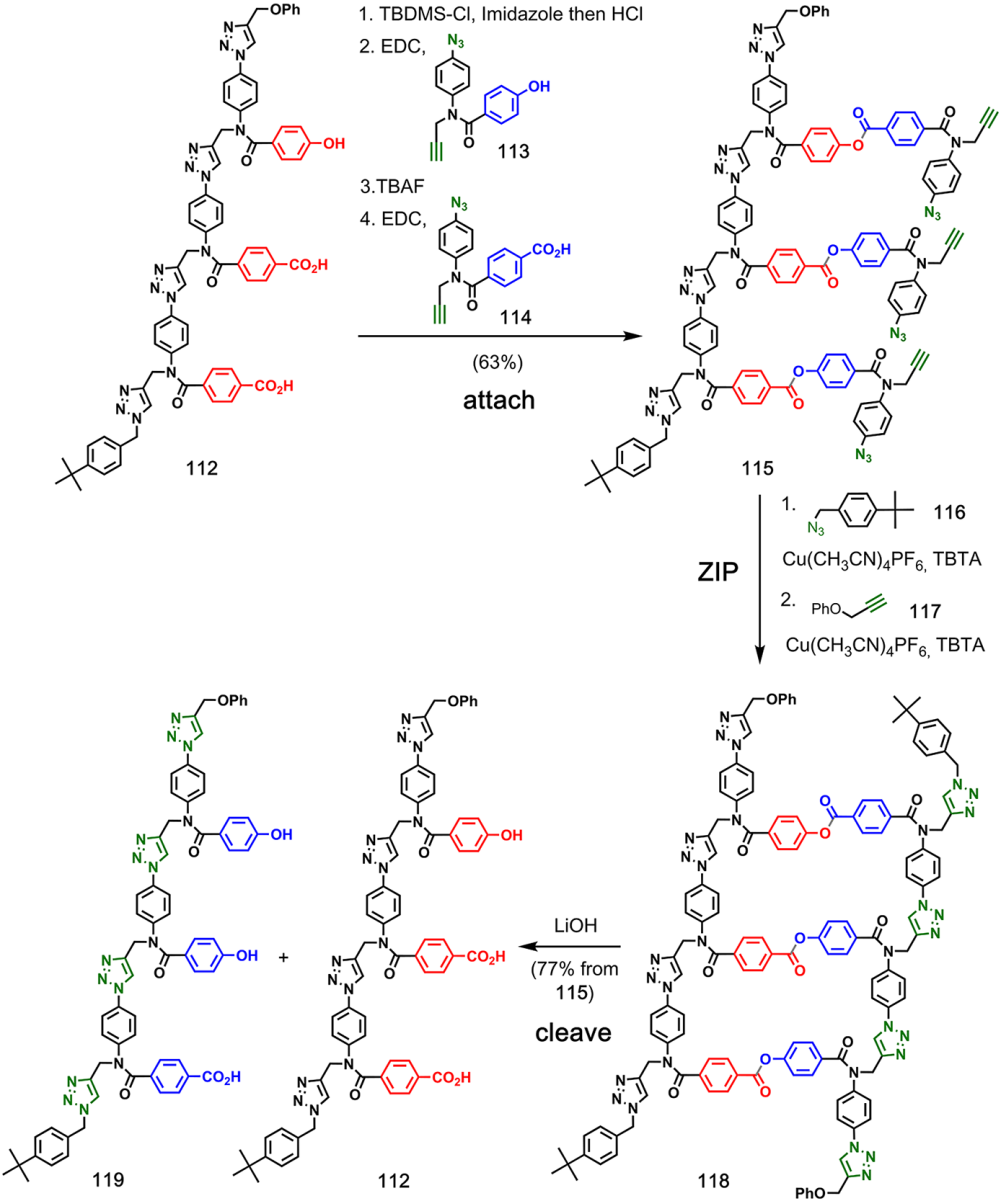
Sequence information transfer using covalent template-directed synthesis based on ester chemistry for the **attach**/**cleave** and CuAAC for the **ZIP** step. Adapted with permission from ref (75). Copyright 2019 Royal Society of Chemistry.

## 3 Mechanically Interlocked Molecules

In the last few decades, the increasing interest in mechanically interlocked molecules, especially in the context of molecular machines, has been accompanied by the development of different template-directed synthetic routes to access them.^47,48^ In these systems, it is not possible to separate the template component from the templated component of the product because they are connected through the mechanical bond that is formed in the templated reaction. We will use the term covalent bond-directed synthesis to distinguish such processes from those where the template is not incorporated into the product.

### 3.1 Ketal Covalent Attachment

The first reported synthesis of an interlocked molecule is Schill’s synthesis of a [2]catenane based on covalent bond-directed synthesis (Figure 12).^16^ In this early example, the formation of ketal **52** takes place in the **attach** step between catechol **50** and ketone **51**, and a subsequent double alkylation of an aniline generates the intra-annularly linked system **53**. Hydrolysis of the ketal and a subsequent oxidation gives [2]catenane **54**. This strategy was later extended to synthesize a [2]rotaxane and [3]catenane.^49,50^

A ketal group was also used by van Maarseveen and co-workers for the synthesis of a [2]catenane (Figure 13).^51^ Two linear strands **55** and **56** equipped with a phenol, alkyne, and alkene functional groups were covalently connected by a central ketal linkage derived from (+)-dimethyl tartrate to produce compound **57**. Then, an aryl diazide temporary linkage (**58**), was attached via transesterification to give the macrocyclic intermediate **59**. The **ZIP** step involved first a CuAAC ring closure of the alkyne strands and then subsequent ring closure of the alkene strands by alkene metathesis. Reduction gave quasi[1]catenane **60**, and the corresponding [2]catenane **61** was obtained in 26% yield after removal of the templates by hydrolysis of the ketal and ester linkages.

### 3.2 Ester Covalent Attachment

Since the early work of Schill, there have been several reports of covalent-bond-directed syntheses of catenanes and rotaxanes. Figure 14 illustrates a somewhat different strategy from the **attach**-**ZIP**-**cleave** pathway discussed above, which was used by Hiratani et al. for the synthesis of [2]rotaxane **66**.^52^ In this case, the macrocycle and axle components of the [2]rotaxane are first connected by ester bonds in the **attach** step between crownophane **62** and succinyl dichloride **63** resulting in compound **64**. Then, aminolysis of the esters in **64** by reaction with two bulky primary amines, such as **65**, gives [2]rotaxane **66** directly. This **trap** step is effectively a concatenation of the **ZIP** and **cleave** steps where the axle is assembled via the formation of two amide bonds, and the ester bonds attaching the axle to the macrocycle are simultaneously cleaved.

**Figure 24:**
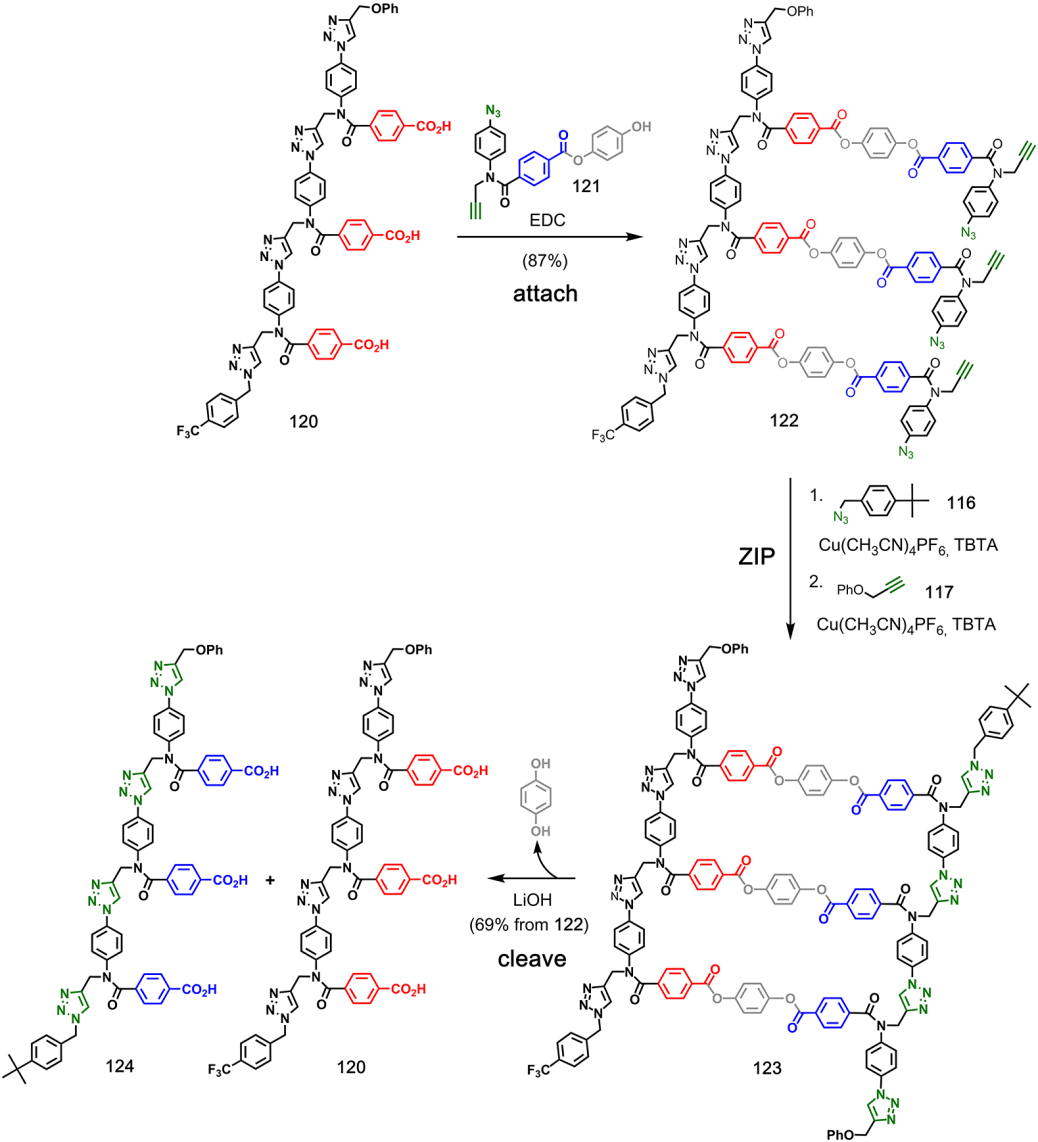
Direct replication of a 3-mer template using covalent template-directed synthesis based on ester chemistry for the **attach**/**cleave** steps and CuAAC for the **ZIP** step. A traceless hydroquinone linker is used to connect two benzoic acid units to yield the identical copy of the template after hydrolysis. Adapted with permission from ref (78). Copyright 2019 Royal Society of Chemistry.

**Figure 25:**
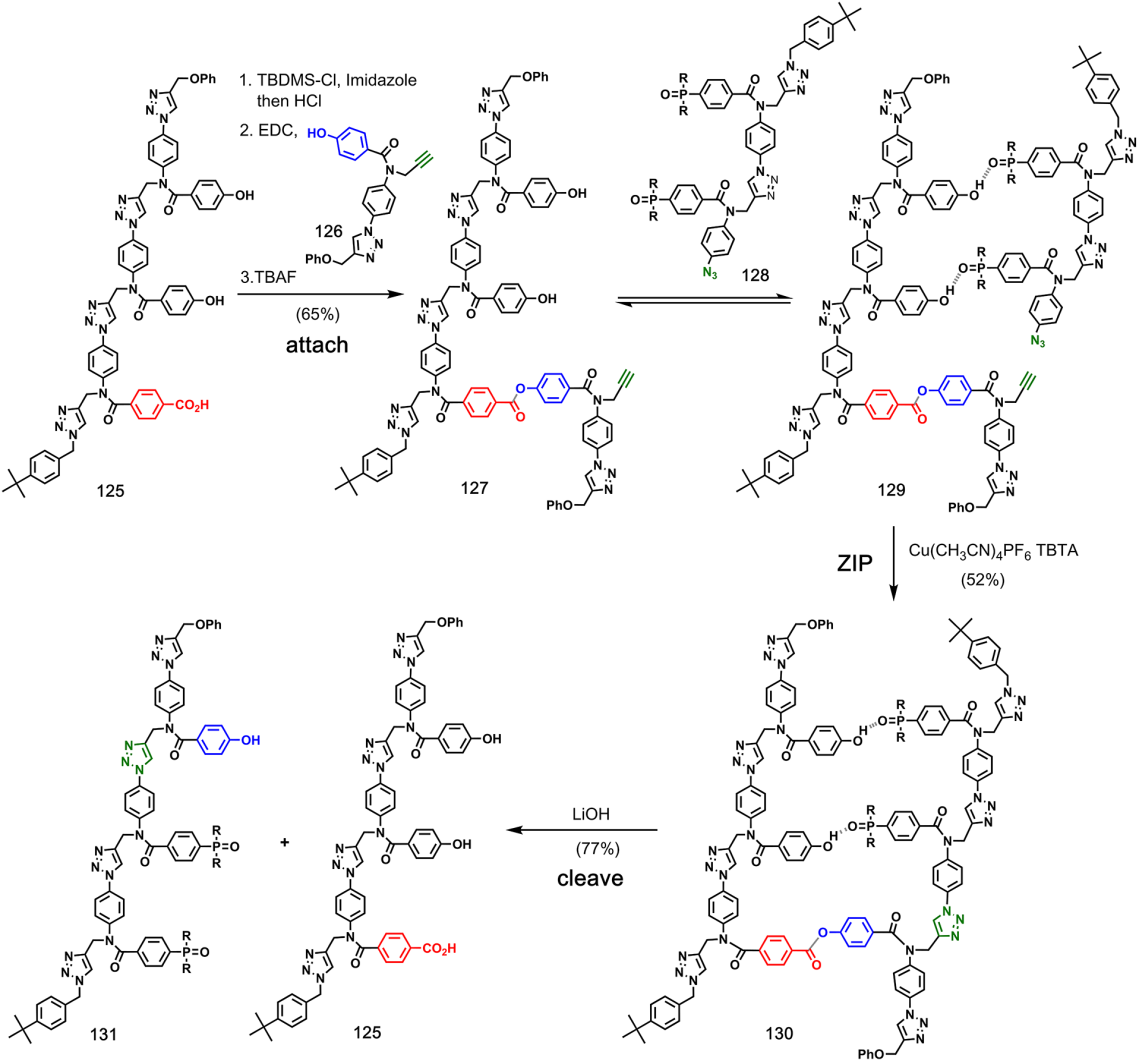
H-bond template-directed oligomer synthesis using a covalent primer, which is first loaded onto the template using sequential template phenol protection, coupling between template benzoic acid and primer, and phenol deprotection. H-bonding drives the selective formation of the hybrid duplex via a CuAAC reaction. Hydrolysis of the primer ester bond releases the copy and regenerates the template. Adapted with permission from ref (80). Copyright 2022 American Chemical Society.

A similar strategy was used in the synthesis of a [1]rotaxane by Hiratani et al. (Figure 15).^53^ The crown ether macrocycle **67**, functionalized with a secondary amine group, was reacted with diacid chloride **68**. Formation of an amide bond and an ester with one of the phenolic hydroxyl groups gave bicyclic compound **69**. Addition of bulky amine **70** led to aminolysis of the ester bond and the formation of [1]rotaxane **71** in 45% yield, together with the noninterlocked product in 20% yield. The same group reported the synthesis of a chiral [2]rotaxane by starting with an asymmetric macrocycle and introducing an asymmetric axle.^54^

A highly selective and high-yielding covalent bond-directed rotaxane synthesis by Tobe and co-workers is shown in Figure 16.^55^ Esterification of phenolic crown ether macrocycle **72** with benzoyl chloride **73** gave prerotaxane **74** in a good yield. Subsequent aminolysis of **74** using amine **75** gave [2]rotaxane **76** in 82% yield.

**Figure 26:**
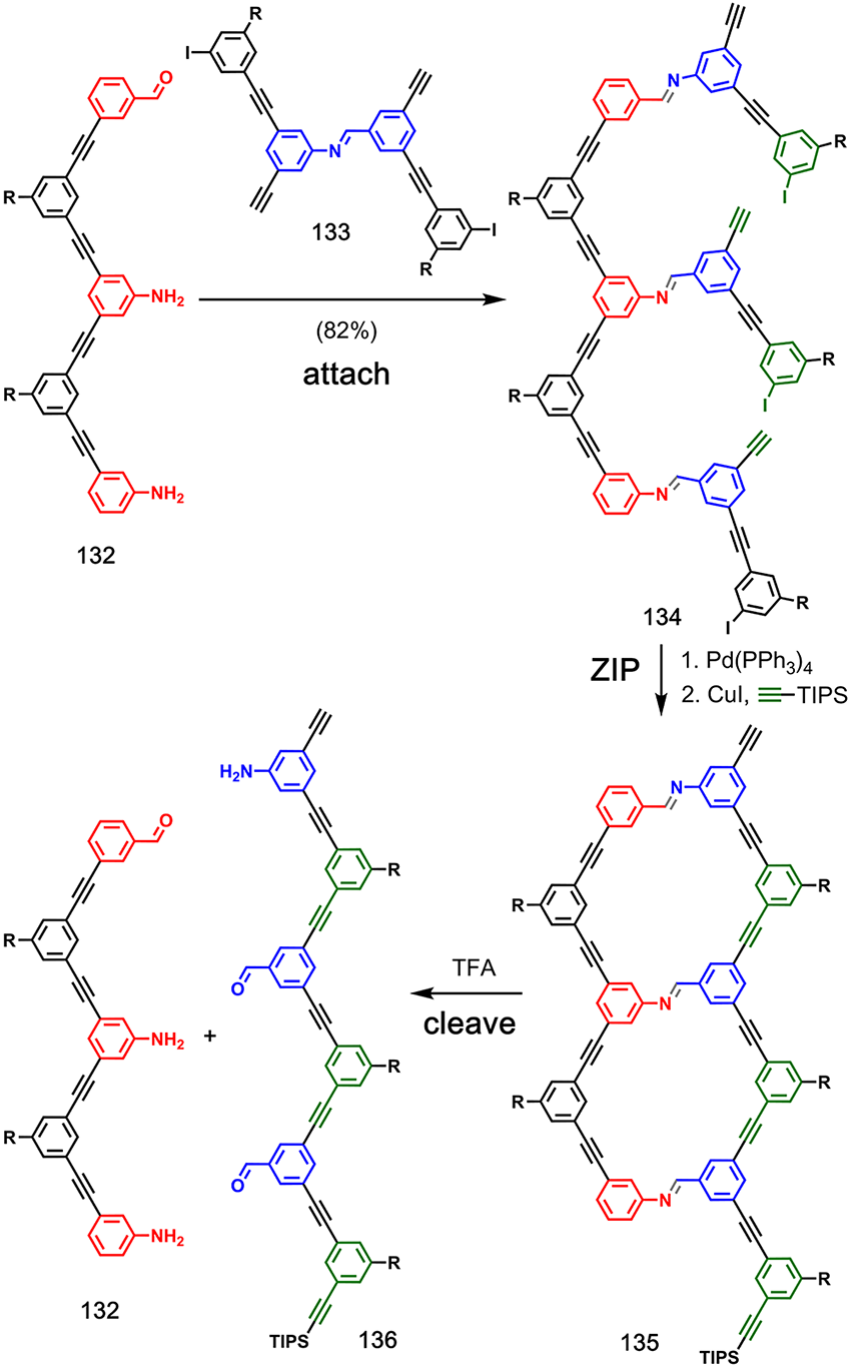
Covalent template-directed oligomer synthesis using imine chemistry in the **attach**/**cleave** step and Sonogashira coupling in the **ZIP** step; R: [2-(2-methoxyethoxy)ethoxy]carbonyl. Adapted with permission from ref (81). Copyright 2024 Wiley.

**Figure 27:**
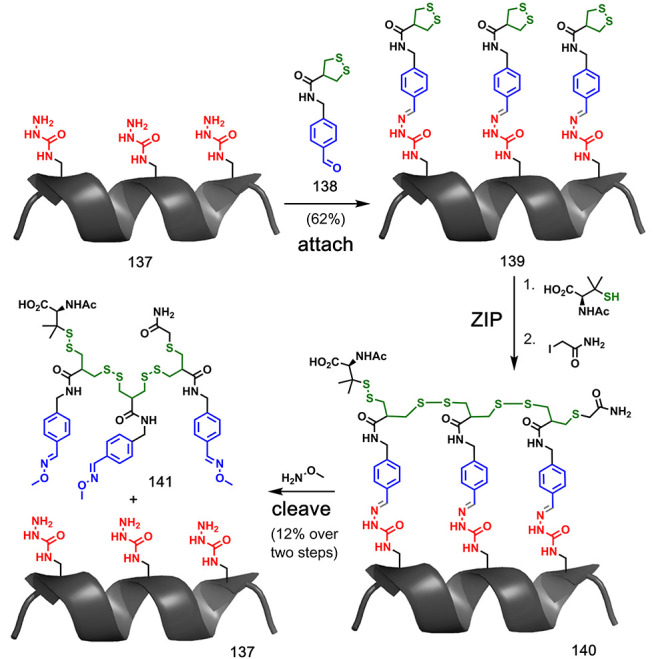
Covalent template-directed oligomerization using imine-based semicarbazone chemistry in the **attach**/**cleave** step and 1,2-dithiolane ring-opening polymerization in the **ZIP** step. Adapted with permission from ref (83). Copyright 2022 Wiley.

**Figure 28:**
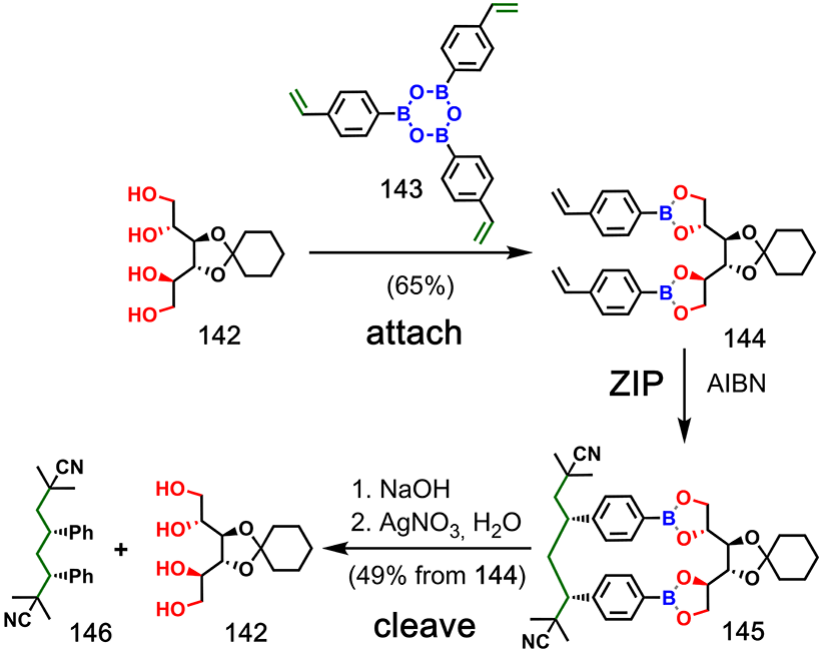
Covalent template-directed dimerization of styrene with stereocontrol using boronic ester chemistry for the **attach**/**cleave** steps and free radical chemistry for the **ZIP** step. Adapted with permission from ref (84). Copyright 1997 American Chemical Society.

Figure 17 shows Höger and co-workers’ synthesis of a phenylacetylene [2]rotaxane.^56^ Two dialkynes **78** were attached to terephthalic acid derivative **77** using ester chemistry to yield the pre-**ZIP** intermediate **79**. Glaser coupling was used to close the macrocycle, which gave access to compound **80** in good yield. Sonogashira coupling was then used to extend the axle with monoprotected diacetylenebenzene **81**. After removal of the alkyne protecting group, macrocycle **82** was used to stopper the axle to give **83**. Finally, the ester bonds were cleaved by aminolysis to give [2]rotaxane **84** in 21% yield. The same diiodo intermediate **80** was also used for the preparation of a [2]catenane by assembling a macrocycle on the two iodo groups and cleaving the ester bonds by aminolysis.^57^

A similar approach was used in the synthesis of a [2]rotaxane by van Maarseveen and co-workers (Figure 18).^58^ Two dialkene building blocks **86** were attached to terephthalate derivative **85** using ester chemistry, and then two bulky stoppers **87** were added using CuAAC. Ring-closing metathesis of resulting pre-**ZIP** intermediate **88** led to macrocycle **89**, and the [2]rotaxane **90** was liberated by transesterification in 54% overall yield. The same authors reported extensions of this strategy to access homo- and hetero[*n*]rotaxanes^59^ and a [2]catenane.^60^ They also published a comprehensive review on covalently templated syntheses of mechanically interlocked molecules.^61^

### 3.3 Miscellaneous

Dynamic covalent chemistry has also been applied to the synthesis of interlocked molecules. Suzuki and co-workers reported the synthesis of a [2]rotaxane directed by temporary imine bonds (Figure 19).^62^ A macrocycle bearing two aniline groups, **91**, was attached to dialdehyde axle **92** by imine formation. Then, two bulky stopper groups were installed in compound **93** via tetra-*n*-butylammonium fluoride (TBAF)-mediated deprotection and alkylation with benzyl bromide derivative **94** to provide prerotaxane **95**. The imine bonds were cleaved by treatment with TFA, and subsequent thioacetalization ensured that [2]rotaxane **96** was irreversibly trapped in 75% yield.

Figure 20 shows the covalent bond-directed synthesis of a [2]catenane consisting of 87-membered rings reported by Godt and co-workers.^63^ In the **attach** step, the macrocyclic carbonate ester **100** was obtained by reacting triphosgene **97** with phenol derivative **98** followed by sodium salt **99**. The **ZIP** step involved a Glaser coupling between terminal acetylenes in **100**, and cleavage of the carbonate ester in compound **101** by hydrolysis gave [2]catenane **102** in 63% yield. This strategy was extended to synthesize analogous [2]catenanes with 63- and 147-membered rings.^64,65^

Itami and co-workers used spirosilylation for covalent template-directed syntheisis of a [2]catenane and a molecular trefoil knot of all-benzene cycloparaphenylene (CPP) rings.^66^ Trichlorosilane **103** was used to covalently connect two molecules of **104** (Figure 21). In the **ZIP** step, Ni(0)-mediated aryl–aryl coupling was used to close the macrocycles at the *para*-chlorobenzene moieties. The Si–C bonds were cleaved with TBAF, and the resulting catenane was subjected to reductive aromatization to provide all-benzene ([12]CPP)([12]CPP)catenane **107**. The same strategy was applied for the synthesis of the ([12]CPP)([9]CPP)catenane and ([9]CPP)([9]CPP)catenane, as well as the [24]CPP trefoil knot.^66,67^ Later, Cong and co-workers described the synthesis of the same ([9]CPP)([9]CPP)catenane using an azobenzene for the covalent attachment of the two components. After a **ZIP** step using aryl–aryl coupling, the **cleave** step involved SmI_2_-mediated reduction of the azobenzene into two aniline moieties, and hydrodeamination of these groups gave the all-benzene catenane.^68^

For the covalent template-directed synthesis of mechanically interlocked molecules, ester chemistry has been most frequently used for attaching substrates to the template. For catenanes, the geometry imposed by functional groups, such as ketals and spirosilanes, has been exploited to organize the intermediate in a suitable conformation for the ZIP step.

## 4 Linear Oligomers

Template-directed synthesis of linear oligomers is more challenging than macrocyclic oligomers because the chain ends of the linear products must be intercepted to prevent formation of macrocycles or higher-molecular-weight intermolecular adducts.

### 4.1 Ester Covalent Attachment

The first reported example of covalent template-directed oligomerization reactions was based on free radical oligomerization of acrylate monomers attached to triphenol template **108** via ester linkages (Figure 22).^69,70^ Intramolecular oligomerization was favored by maintaining very low concentration in the reaction mixture throughout the reaction by the slow addition of a solution of the triester **109** and a solution of azobisisobutyronitrile (AIBN) to a large volume of solvent. 2-Cyanoprop-2-yl radicals acted as both the radical initiator and the chain terminator to cap the oligomer ends and prevent intermolecular reactions. The templated product **110** was obtained in 70% yield, and cleavage by basic hydrolysis released pentacarboxylic acid **111** from template **108**. Use of the corresponding diphenol template yielded a tetracarboxylic acid. Replacing the methylene bridges between the phenol moieties of the template with sulfones resulted in similar templating efficiency.^71^

Linear templating is the basis of nucleic acid replication where a linear template encoding sequence information is copied into a linear daughter strand.^1,2^ This process is the molecular foundation for the evolution of living organisms. Over the last decades, the development of *in vitro* molecular evolution has offered a convenient tool for the optimization of existing biopolymers for therapeutic or manufacturing applications and the synthesis of new functional biopolymers.^72−74^ Attempts to develop similar techniques for synthetic oligomers require a method to replicate sequence information in synthetic oligomers, and covalent template-directed synthesis is a promising approach. Hunter and co-workers have developed a method for sequence information transfer between synthetic triazole oligomers, which is based on the use of ester chemistry to attach monomers to a template (Figure 23).^75^ Information was encoded in template **112** as the sequence of phenol and benzoic acid side chains, and two different monomers, **113** and **114**, were attached to the complementary groups via a series of high-yielding reactions: selective protection of the phenol side-chain, coupling of the benzoic acid side-chains with phenol monomers, and deprotection and coupling of the phenol side-chain with the benzoic acid monomer. The monomers contain an azide and an alkyne group; therefore, CuAAC could be used in the **ZIP** step from intermediate **115** to obtain the covalent duplex **118**. The capping azide **116** was used *in situ* to intercept the terminal alkyne from competing macrocyclization and intermolecular reactions, and the terminal azide was then capped with alkyne **117**.^76,77^ Cleavage of the esters regenerated template **112** and released complementary copy **119**.

Hunter and co-workers also reported the use of traceless linkers to attach monomers to the template so that either direct replication, reciprocal replication, or mutation of sequence information can be achieved.^78,79^ The base-pairing system described in Figure 23 leads to a reciprocal copying method analogous to nucleic acid replication. Figure 24 shows how incorporation of traceless linkers to connect two carboxylic acids leads to direct replication of a sequence-identical copy of the template.^78^ The first step involved the attachment of monomer **121** bearing the traceless linker to template **120** via ester coupling. Then, intramolecular CuAAC reactions of **122** in the presence of capping azide **116** followed by reaction with **117** yielded duplex **123**. Hydrolysis of the ester moieties regenerated triacid template **120**, along with triacid copy **124**. The traceless linker base-pairing strategy can also be used to introduce mutations in the replication process.^79^ By spiking the symmetrical base pairs used in direct replication with unsymmetrical base pairs that lead to reciprocal replication, mutations can be implemented at a rate directly determined by the proportions of the different monomers used in the **attach** step.

Hunter and co-workers reported a hybrid approach that uses a combination of covalent bonds and noncovalent interactions to attach reactants to the template (Figure 25).^80^ In the first step, phenol monomer **126** was attached to a benzoic acid side chain on template **125** using ester chemistry. The phenol side chains on the template were then used to bind phosphine oxide oligomer **128** to primed template **127** via H-bonding interactions. A CuAAC **ZIP** step gave templated product **130**, which was cleaved by hydrolysis of the ester linkage to provide template **125**, along with complementary copy **131**.

### 4.2 Imine and Related Covalent Attachment

Szostak and co-workers reported template-directed synthesis of linear oligomers using dynamic covalent chemistry for the **attach** step (Figure 26).^81,82^ Formation of imine bonds between aniline and benzaldehyde groups was used to attach monomers to template **132**. The resulting pre-**ZIP** intermediate **134** was subjected to intramolecular Sonogashira coupling under dilute conditions to give the duplex **135**. TFA-mediated cleavage of the imine linkages regenerated template **132**, along with a templated copy strand, which was shown to contain one aniline and two benzaldehyde monomers and presumably has the sequence shown in **136**.

**Figure 29:**
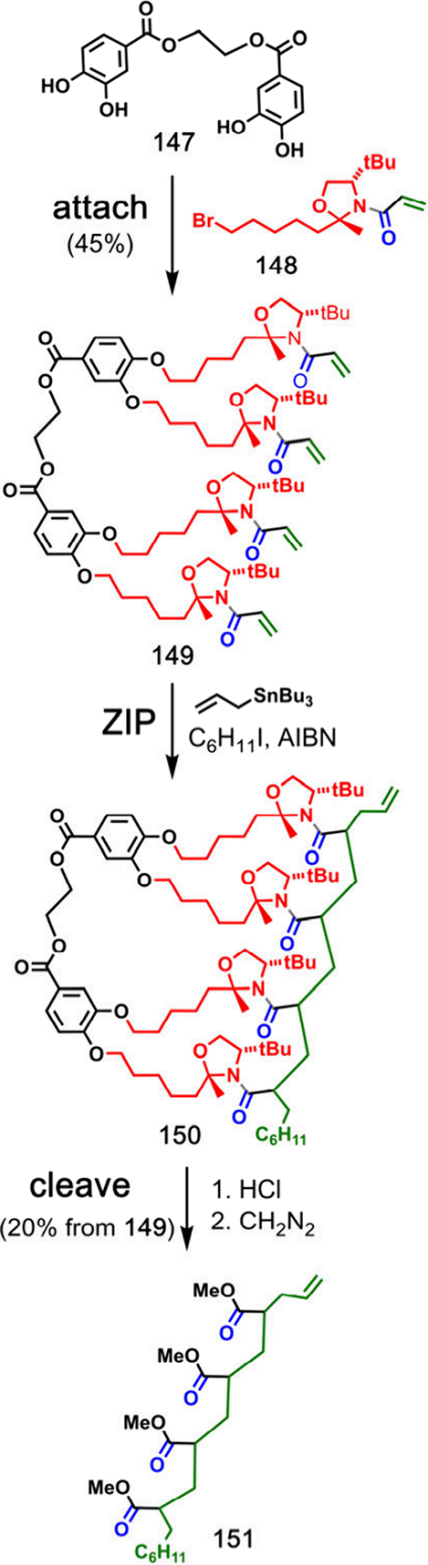
Covalent template-directed free radical oligomerization of *N*-acrylamide monomers. Adapted with permission from ref (87). Copyright 2000 Wiley.

**Figure 30:**
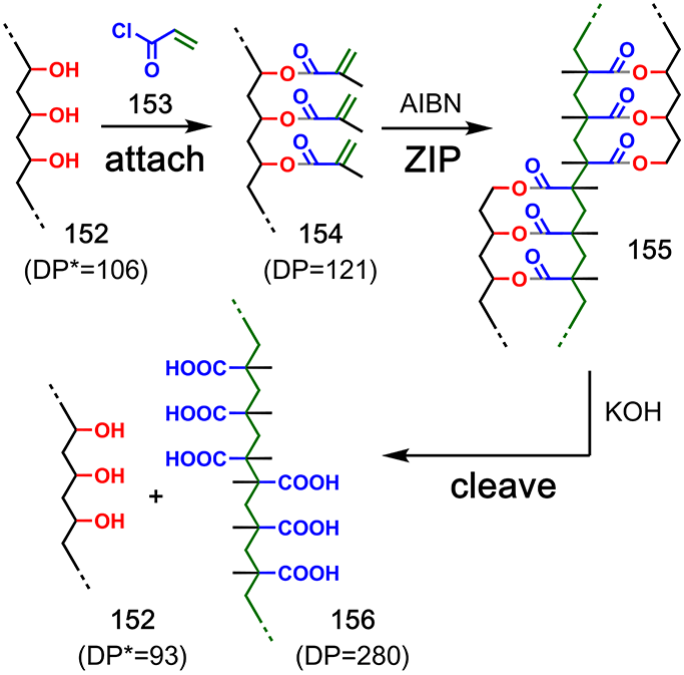
Intermolecular reactions between chains lead to high-molecular-weight products in the covalent template-directed polymerization of methacrylic acid on a polyvinyl alcohol template. DP* values were determined from *M*_v_, therefore indicating an upper limit on DP. Adapted with permission from ref (88). Copyright 1986 Wiley.

**Figure 31:**
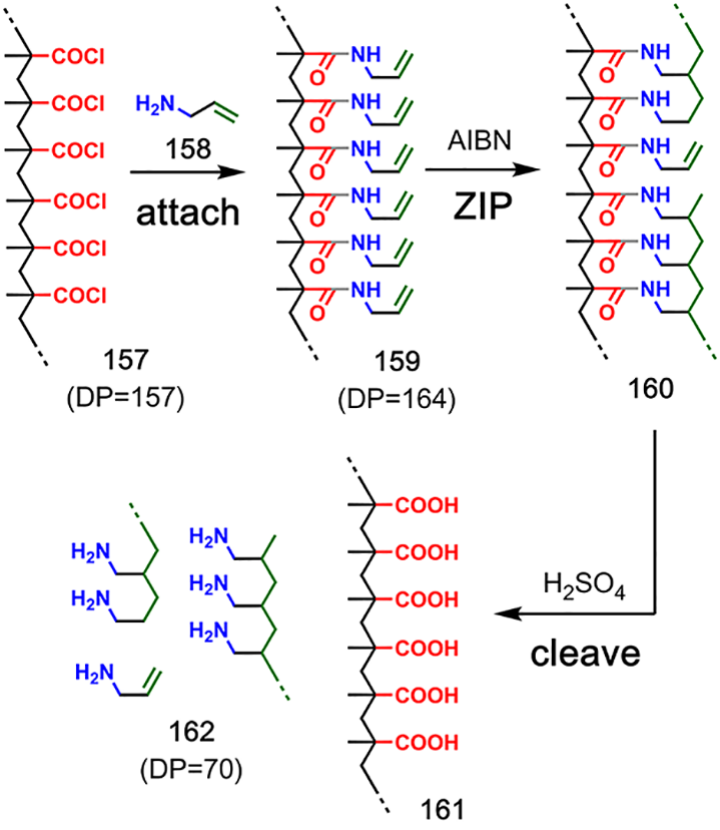
Incomplete intramolecular coupling reactions lead to low-molecular-weight products in the covalent template-directed polymerization of allylamine on a polymethylmethacroyl chloride template. Adapted with permission from ref (89). Copyright 1989 Wiley.

Dynamic covalent chemistry combined with ring-opening polymerization is the basis of the templated oligomerization methodology developed by Matile and co-workers.^83^ A 3_10_-helical peptide with three semicarbazide groups on the same face of the helix (**137**) was used as the template (Figure 27). In the **attach** step, aldehyde **138** bearing a 1,2-dithiolane moiety was loaded onto the template to give semicarbazone derivative **139**. Ring-opening polymerization of the 1,2-dithiolane groups was employed in the **ZIP** step using a thiol initiator and iodoacetamide as the end-capping group. The resulting duplex **140** was cleaved with methoxyamine to give templated product **141** in relatively low yield (12%).

### 4.3 Miscellaneous

Boronic ester chemistry has been successfully applied to the **attach**/**cleave** steps in the stereoselective template-directed synthesis of linear oligomers. Enantiopure template **142**, which is a monoacetal of mannitol, was first functionalized with vinyl monomers by using boronic ester linkages (Figure 28). The product **144** was subjected to free radical polymerization with AIBN.^84^ After hydrolysis and deborylation of duplex **145**, the 2-cyanoprop-2-yl-capped product **146** was isolated. Following quantification of the different stereoisomers by gas chromatography and chiral HPLC, the major stereoisomer was found to have formed with 4.8% de and 85.7% ee, indicating good enantioselectivity of the templating step.

Porter and co-workers reported covalent template-directed free radical polymerization of *N*-acrylamide monomers (Figure 29).^85−87^ Acrylic acid monomers **148** were attached to polyol template **147** via oxazolidine linkers to yield intermediate **149**. By adjusting the concentration of the chain transfer reagent (allyltributyltin), the yield of the templated product **150** could be optimized. Hydrolysis cleaved the oligocarboxylic acid product from the template, and reaction with diazomethane converted this to the corresponding oligoester **151**. A dimer template gave 87% of the dimer product, and a tetramer template (Figure 30) gave 20% of the tetramer product, which is quite different from the product distributions in nontemplated polymerization reactions.^86,87^ The template was not recovered after the **cleave** step.

**Figure 32:**
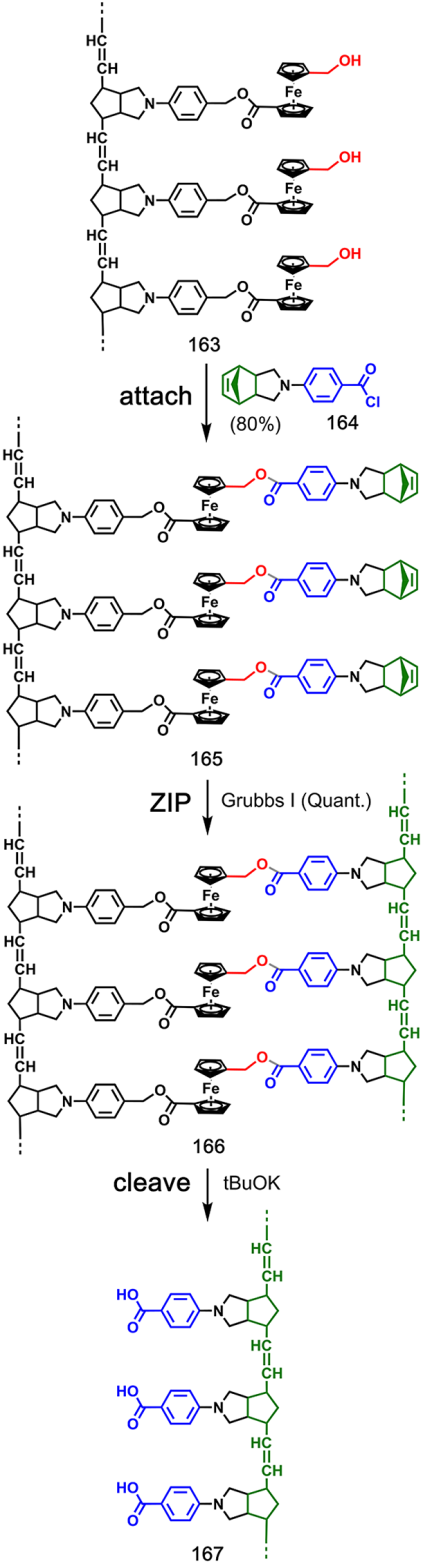
Covalent template-directed ring-opening metathesis polymerization of norbornene monomers on a polynorbornene template. The template was not recovered in the **cleave** step. Adapted with permission from ref (90). Copyright 2007 Wiley.

**Figure 33:**
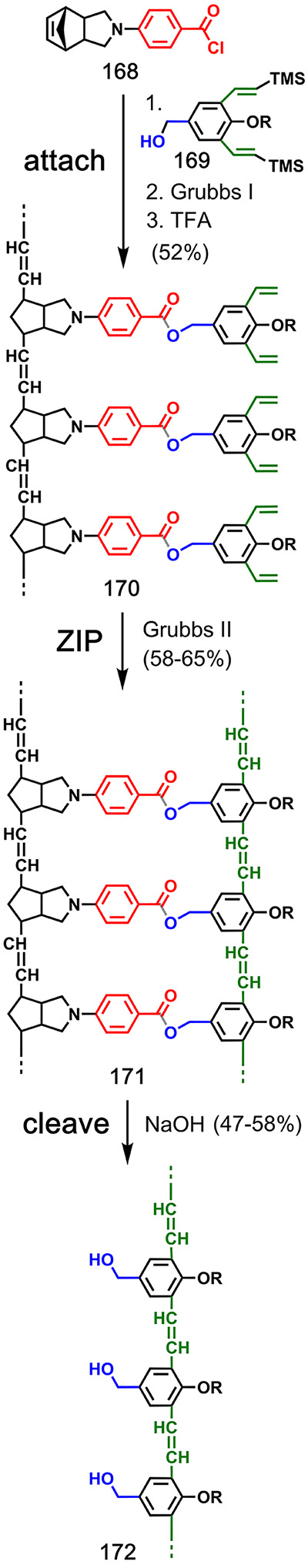
Covalent template-directed ring-closing metathesis polymerization of divinylbenzene monomers on a polynorbornene template (R: *n*-C_12_H_25_). Adapted with permission from ref (91). Copyright 2013 American Chemical Society.

Both ester chemistry and dynamic covalent approaches have been successfully exploited for the covalent template-directed synthesis of linear oligomers. One of the key features determining the efficiency of these processes is the flexibility of the template because undesired macrocyclization reactions compete with the formation of linear chains. The examples highlighted in this section show that the compatibility of ester and imine attach chemistry with different **ZIP** reaction conditions make them ideal candidates for further development.

## 5 Polydisperse Polymers

Covalent template-directed synthesis has been successfully used to control the average molecular weight of polydisperse homopolymers. An early example involved attachment of methacroyl chloride **153** to polyvinyl alcohol template **152** (Figure 30).^88^ Free radical polymerization of the methacrylate groups in the resulting compound **154** was used for the **ZIP** step, and then, the ester linkages in the product **155** were cleaved by basic hydrolysis. The polyvinyl alcohol **152** was recovered with a degree of polymerization (DP) similar to the original template (approximately 110). However, poly(methacrylic acid) product **156** had a DP of 280, which suggested that the major product of the templated reaction was a dimer of the desired duplex, which is formed either by propagation of the radical chain reaction from one template-bound chain to a second one or by termination via chain recombination (Figure 30).

**Figure 34:**
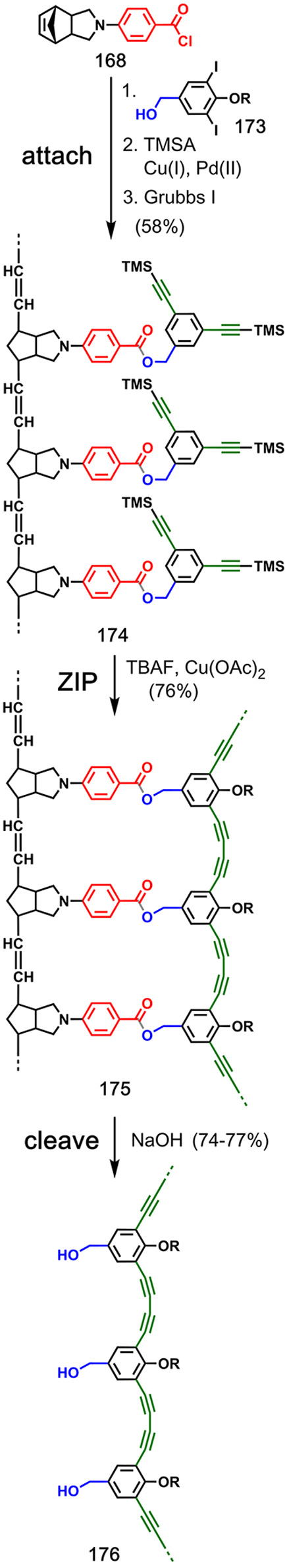
Covalent template-directed polymerization diethynylbenzene monomers by Glaser coupling on a polynorbornene template (R: *n*-C_16_H_33_). Adapted with permission from ref (92). Copyright 2011 Wiley.

**Figure 35:**
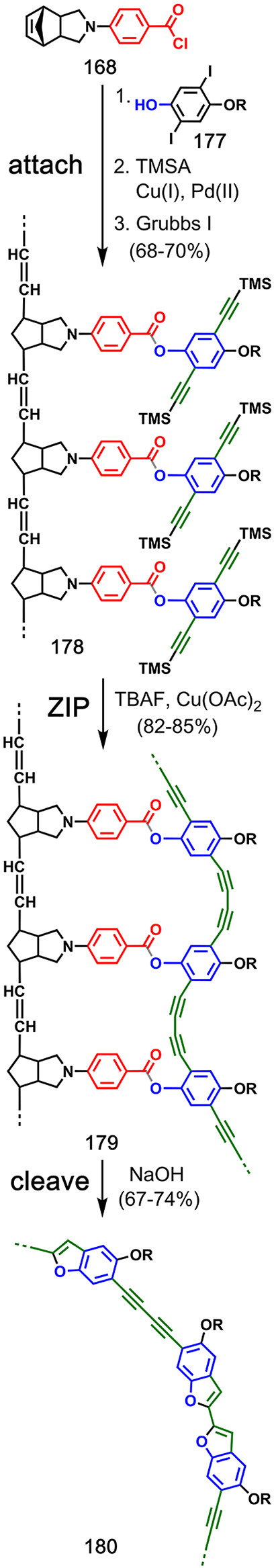
Covalent template-directed polymerization of diethynylbenzene monomers by Glaser coupling on a polynorbornene template. The alkynyl moieties can couple in three different ways so the templated product is a mixture of isomers, and annulation reactions take place in the **cleave** step leading to a polybenzofuran product (R: *n*-C_12_H_25_). Adapted with permission from ref (91). Copyright 2013 American Chemical Society.

The same authors also attempted to control the polymerization of allylamine **158** using polymethylmethacroyl chloride **157** as a template (Figure 31).^89^ Formation of amide bonds was used to attach the monomers to the template to form pre-**ZIP** intermediate **159**, and then, free radical polymerization was initiated using AIBN. Cleavage of the amide linkages in **160** under acidic conditions regenerated the template derivative **161** along with the polyallylamine product **162**. In this case, the DP of the template was 160, but the DP of the polyallylamine obtained was 70. Degradative chain transfer in the **ZIP** step was proposed as a possible cause of this discrepancy. These early examples highlight the difficulties involved in templating the synthesis of linear oligomers. Coupling between monomers that are not at adjacent positions on the template chain will lead to products with a DP that is significantly lower than that of the template, and intermolecular reactions between chains will lead to products with a DP that is significantly higher. Methods to minimize these processes are, therefore, required for efficient templating of polymerization reactions.

**Figure 36:**
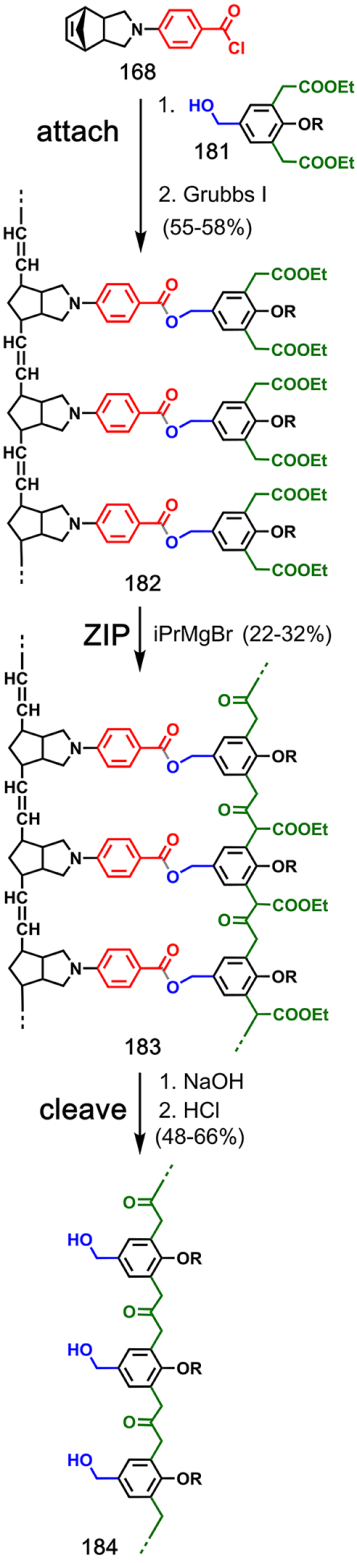
Covalent template-directed polymerization of diester monomers by Claisen ester condensation on a polynorbornene template. The esters can couple in three different ways, but decarboxylation in the **cleave** step removed the remaining esters to give a product with a uniform polyketone backbone (R: *n*-C_12_H_25_). Adapted with permission from ref (91). Copyright 2013 American Chemical Society.

Luh and co-workers achieved the first successful covalent templated polymerizations using different types of monomers attached to a polynorbornene template via ester chemistry (**164** to template **163** in Figure 32; **169** to **168** in Figure 33; **173** to **168** in Figure 34; **177** to **168** in Figure 35; **181** to **168** in Figure 36).^90−92^ The substrates for the **ZIP** reactions were prepared by ring-opening metathesis polymerization (ROMP) of functionalized norbornenes (**165** in Figure 32; **170** in Figure 33; **174** in Figure 34; **178** in Figure 35; **182** in Figure 36), which gave narrow polydispersity (PDI = 1.1–1.4, as determined by GPC). Multiple samples differing in template DP were prepared by using different norbornene monomer-to-catalyst ratios (DP = 10–25). The templated **ZIP** step was carried out at concentrations of 0.5–14 mM using ring-opening or ring-closing metathesis polymerization (Figures 32 and 33),^90,91^ Glaser coupling (Figures 34 and 35),^91,92^ or Claisen ester condensation (Figure 36).^91^ The resulting duplexes were cleaved by ester hydrolysis, and the PDI and DP of the templated products were determined by end-group analysis and GPC (**166** to **167** in Figure 32; **171** to **172** in Figure 33; **175** to **176** in Figure 34; **179** to **180** in Figure 35; and **183** to **184** in Figure 36). In all cases, the DP of the copy strand closely resembled the DP of the template, and increasing the length of the template resulted in a copy with an increased DP (Table 2). Similarly, the PDI of the copy strands were similar to the PDI of the templates. Untemplated polymerizations of the corresponding monomer units were also carried out. Higher concentrations were generally required (5–233 mM), and the resulting polymers had increased polydispersity (PDI = 2.2–2.6) compared with the templated reactions. The control over PDI and DP in the templated reactions, together with successful polymerization at low concentrations, provides good evidence for a significant template effect in these systems.

**Table 2:** DP of Template and Product Polymers Synthesized by Luh and Co-workers^a^

chemistry	template/pre-ZIP		duplex		copy	
	compd	DP	compd	DP	compd	DP
Ring-opening metathesis (Figure 32)	**163**	20	**166**		**167**	18
Ring-closing metathesis (Figure 33)	**170**	11	**171**	12	**172**	11
		15		14		15
		26		26		23
Glaser coupling (Figure 34)	**174**	11	**175**	10	**176**	11
		14		12		14
		18		16		17
Glaser coupling (Figure 35)	**178**	10	**179**	9	**180**	10
		15		16		15
Claisen ester condensation (Figure 36)	**182**	7	**183**	7	**184**	7
		12		12		11
		15		15		13

a DP values shown were determined by GPC.

The structural requirements for the backbone and covalent attachment chemistry required for successful templated polymerization reactions that result in polydisperse linear polymers are identical to those for discrete linear oligomers: the polymeric template strand should have sufficient rigidity to favor linear over macrocyclic products. The benzoate ester moieties used by Luh and co-workers appear to be suitable functional groups for efficient templating. Considering the successful application of imine-based chemistry in the preparation of discrete linear oligomers, this chemistry might be a useful approach for further developments in the covalent template-directed synthesis of polydisperse polymers.

## 6 Cross-Linked Polymer Networks

**Figure 37:**
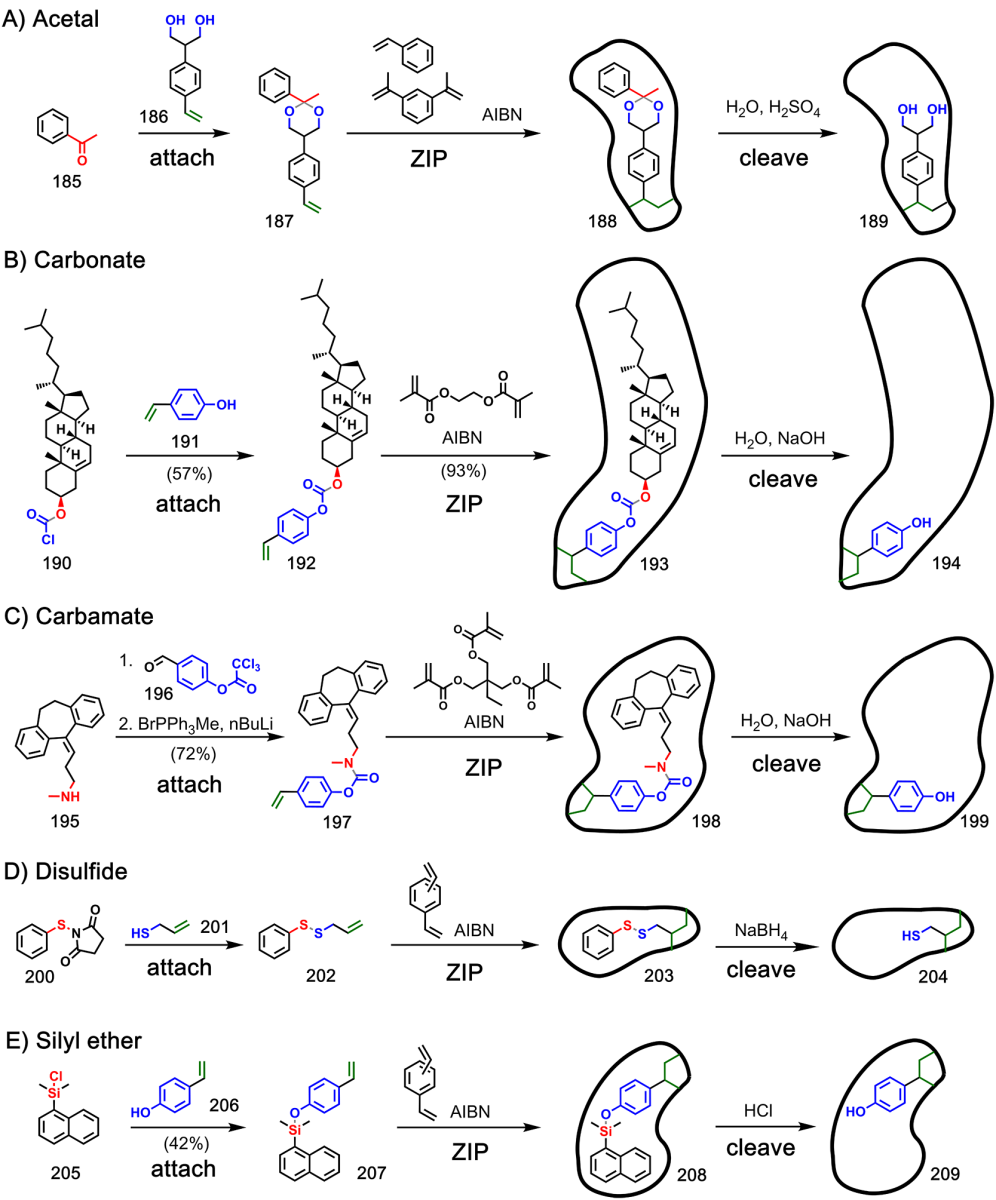
Examples of molecular imprinting using templates covalently attached to one polymerizable monomer via (A) acetal (Adapted with permission from ref (96). Copyright 1991 American Chemical Society.), (B) carbonate ester (Adapted with permission from ref (97). Copyright 1995 American Chemical Society.), (C) carbamate (Adapted with permission from ref (98). Copyright 2001 Elsevier.), (D) thiol (Adapted with permission from ref (99). Copyright 2003 Elsevier.), and (E) silyl ether linkages (Adapted with permission from ref (101). Copyright 2000 Elsevier.).

**Figure 38:**
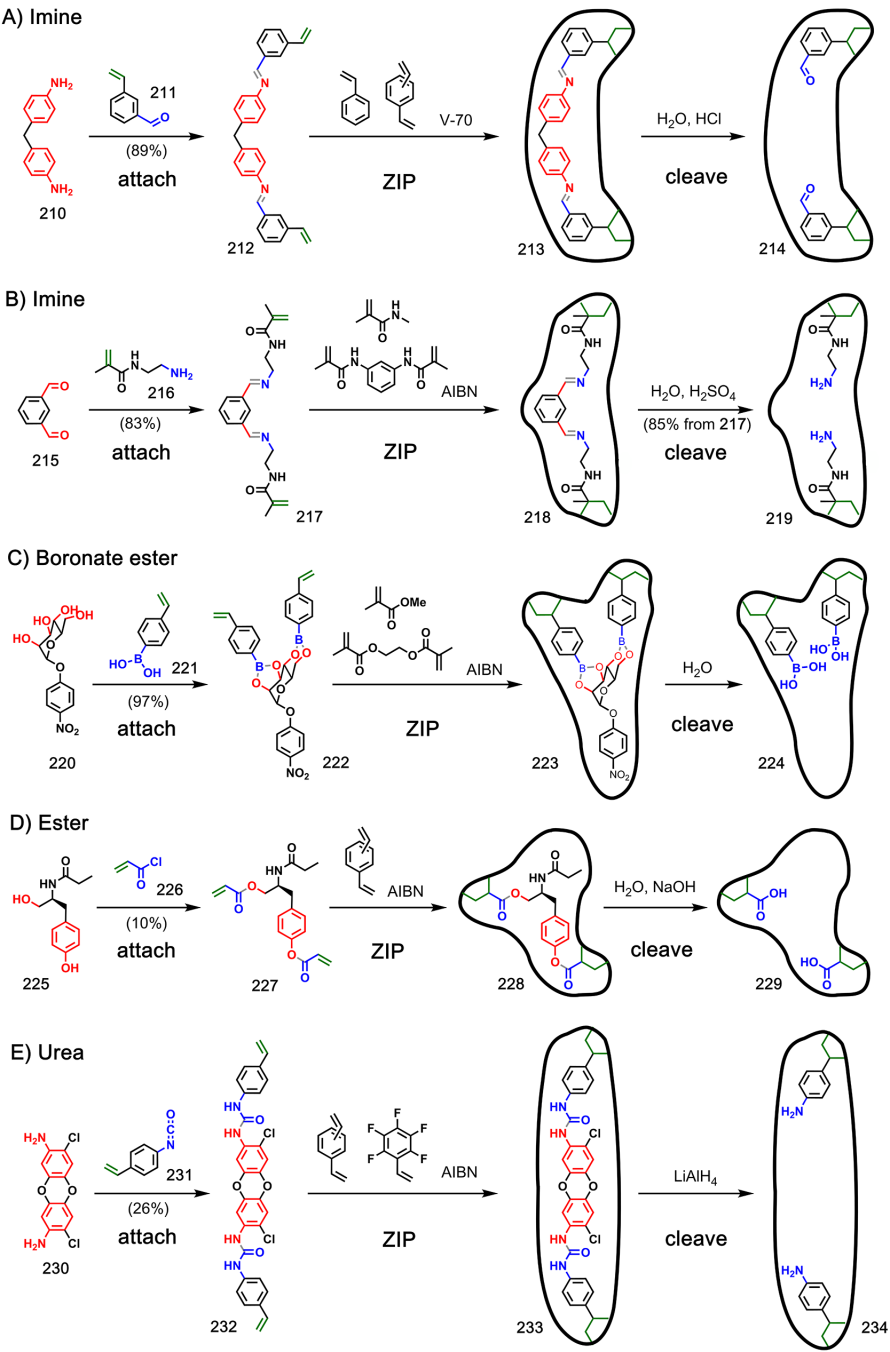
Examples of molecular imprinting using templates covalently attached to two polymerizable monomers via (A) imine (Adapted with permission from ref (107). Copyright 2012 Taylor & Francis.), (B) imine (Adapted with permission from ref (108). Copyright 1990 American Chemical Society.), (C) boronate ester (Adapted with permission from ref (109). Copyright 1977 Wiley.), (D) ester (Adapted with permission from ref (111). Copyright 1990 American Chemical Society.), and (E) urea linkages (Adapted with permission from ref (112). Copyright 1998 American Chemical Society.).

**Figure 39:**
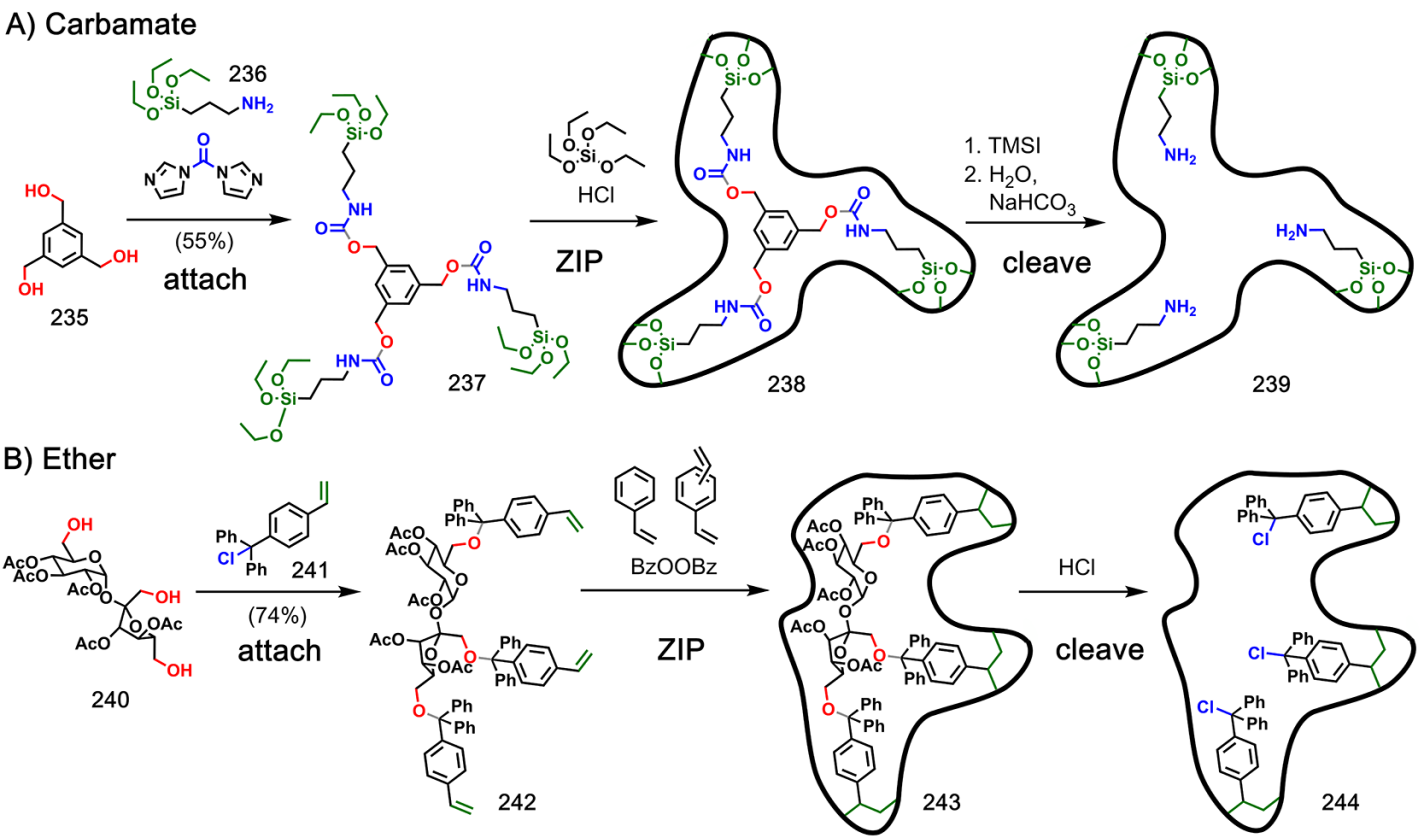
Examples of molecular imprinting using templates covalently attached to three polymerizable monomers via (A) carbamate (Adapted with permission from ref (117). Copyright 2000 Macmillan Magazines Ltd.) or (B) ether linkages (Adapted with permission from ref (118). Copyright 1996 Elsevier.).

Both covalent and noncovalent templating have been used to control the size and functionality present in cavities in cross-linked polymer networks in a process called molecular imprinting. This field has been the subject of numerous reviews,^93−95^ so here, we present a brief summary of the different types of covalent bonds that have been used to attach polymerizable monomers to templates (Figures 37–39). In each case, polymerization was carried out in the presence of a cross-linking agent to give polymer monoliths that incorporate the template in cavities throughout the material. Cleavage and removal of the template yield a macromolecule that has cavities that are complementary to the template. The ability of the template molecules to rebind in the polymer is usually demonstrated by affinity chromatography or incubation of the polymer with the template and quantification of the unbound template in solution. In most cases, it has been shown that rebinding of the template is favored over closely related structural analogues, thereby providing good evidence for a significant template effect in these systems.

Figure 37 shows examples where one polymerizable monomer was attached to a template via an acetal (**185**–**189**),^96^ carbonate ester (**190**–**194**),^97^ carbamate (**195**–**199**),^98^ thiol (**200**–**204**)^99,100^ or silyl ether linkage (**205**–**209**).^101^ In addition to those shown in Figure 37, there are examples of molecular imprinting using templates covalently attached to one polymerizable monomer via a boronate ester,^102^ ester,^103−105^ or thiocarbamate.^106^Figure 38 shows examples where two polymerizable monomers were attached to a template via an imine (**210**–**214** and **215**–**219**),^107,108^ boronate ester (**220**–**224**),^109,110^ ester (**225**–**229**),^111^ or urea linkage (**230**–**234**).^112^ Carbamate chemistry^113,114^ and simultaneous use of amide and disulfide bonds^115,116^ have also been exploited in molecular imprinting where two different polymerizable monomers were attached to a template. Figure 39 shows examples where three polymerizable monomers were attached to a template via a carbamate (**235**–**239**)^117^ or ether linkage (**240**–**244**).^118^ Note that some of these linkages have been deployed in two different ways to give different functionality in the cavities of the imprinted polymer product. For example, imine linkages have been used to generate amines or aldehydes in the product (Figure 38), and carbamate linkages have been used to generate alcohols or amines in the product (Figures 37 and 39).

**Figure 40:**
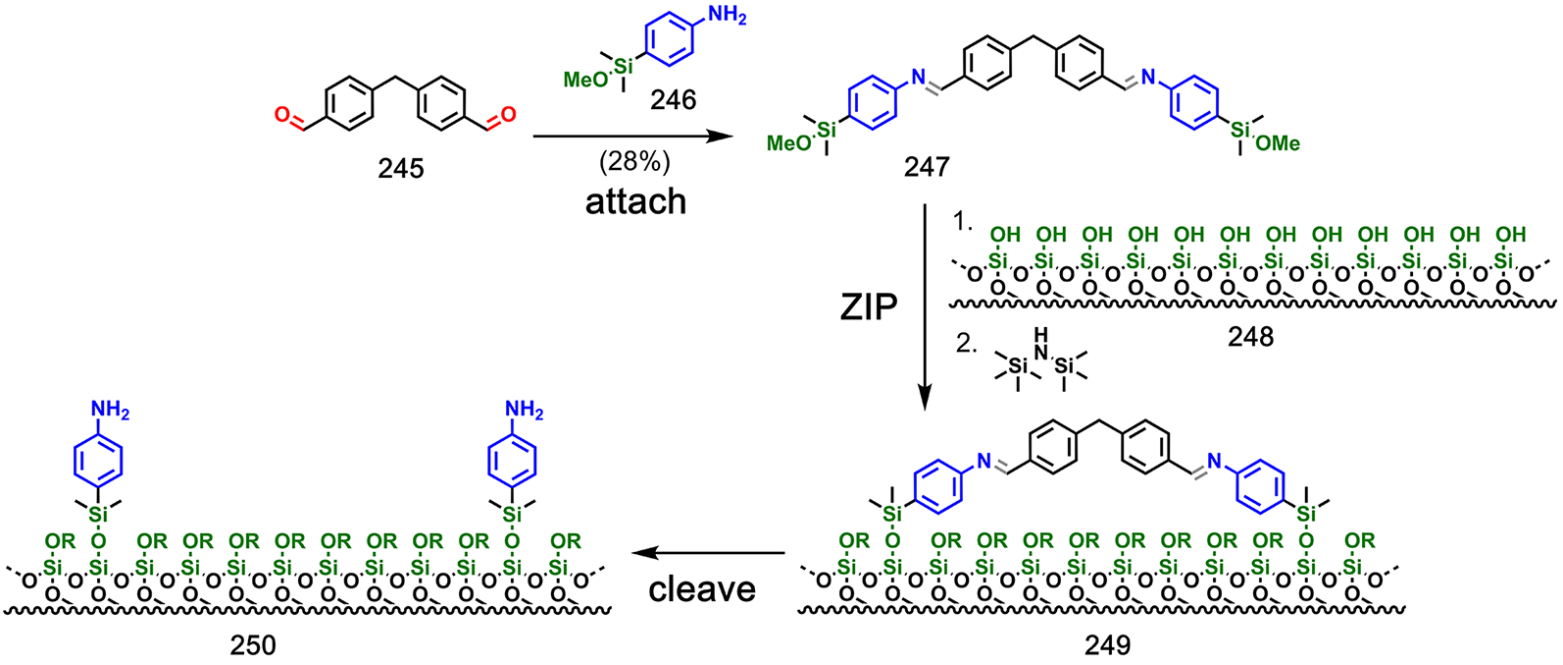
Covalent template-directed functionalization of a silica surface with aniline groups using imine chemistry in the **attach**/**cleave** steps where OR represents octadecyltrimethoxysilane (OTMS) with some remaining unfunctionalized OH groups. Adapted with permission from ref (119). Copyright 1986 American Chemical Society.

In addition to three-dimensional materials, molecular imprinting has also been used to functionalize two-dimensional surfaces. Figure 40 shows covalent template-directed functionalization of a silica surface with aniline groups.^119^ The aniline **246** was attached to the dialdehyde template **245** via imine chemistry. The resulting compound **247** was subjected to the formation of silyl ethers with a silica surface **248** in the **ZIP** step to provide chemically modified surface **249**. Cleavage of the imines resulted in surface **250** that has pairs of aniline groups at a well-defined separation. By using dialdehyde templates of different lengths, it was possible to create functionalized surfaces with different spacings between the aniline groups. The resulting surfaces were able to rebind the complementary dialdehyde template with greater affinity than surfaces created with shorter or longer dialdehydes.

**Figure 41:**
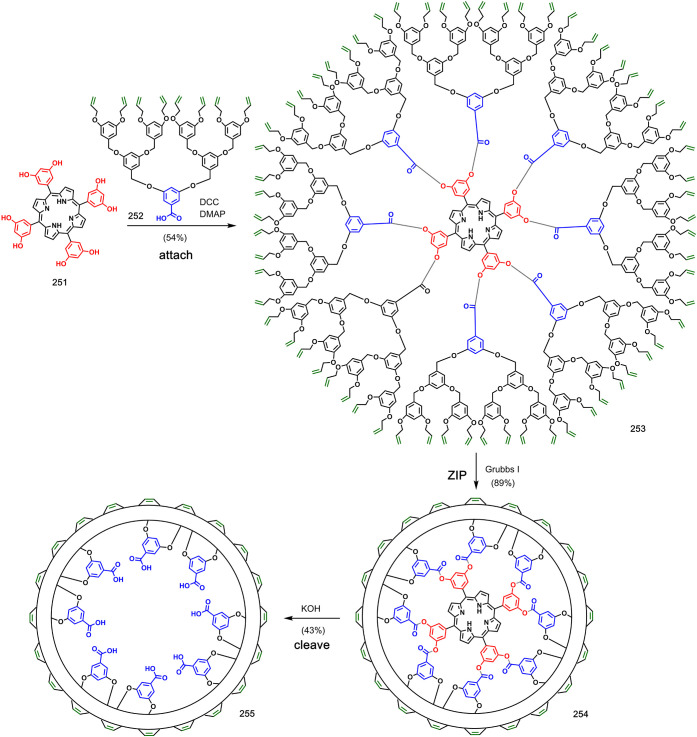
Cored dendrimers synthesized by covalent template-directed ring-closing metathesis polymerization. Adapted with permission from ref (121). Copyright 2002 Macmillan Magazines Ltd.

Covalent template-directed molecular imprinting has also been employed in the synthesis of soluble polymer nanoparticles (Figure 41).^120−122^ Ester coupling was used to attach dendrimer **252** bearing eight terminal alkenes to porphyrin template **251**. Intramolecular ring-closing metathesis reactions of pre-**ZIP253** afforded product **254** as a discrete molecule. Cleavage of the esters by hydrolysis and removal of the template gave products **255**, which were dubbed cored dendrimers. The cavity in the center of the polymacrocyclic product shown in Figure 41 had a high affinity for the original porphyrin template.^121^

The examples included in this section highlight the variety of **attach**/**cleave** reactions available for efficient covalent template-directed molecular imprinting. In contrast to the examples above, here, the template effect is related to the shape of polymer network formed around the guest molecules, which is usually quantified by investigating the noncovalent rebinding of the template to the imprinted polymer. The outcome of templated cross-linked polymerization reactions is affected by the precise reaction conditions used in addition to the chemical structure of the pre-**ZIP** intermediate. This feature broadens the list of suitable reactions for the **attach**/**cleave** steps, as illustrated in Figures 37–39, but ester and imine-based chemistry remain a popular choice for the development of functional materials via covalent template-directed synthesis.

## 7 Conclusions and Future Perspectives

In the last 60 years, template-directed synthesis has become a powerful synthetic methodology to access complex molecules that are difficult to synthesize by other routes. The attachment of molecular building blocks to a template enables a particular geometric arrangement of the reactive groups, lowering the transition-state energy and facilitating a particular reaction pathway over unwanted alternatives. The use of noncovalent interactions to connect the template and substrates has been widely explored in supramolecular chemistry, especially for the preparation of macrocycles and mechanically interlocked molecules. Nevertheless, there are some limitations in noncovalent template-directed synthesis. The substrates and template must be fully assembled in the pre-**ZIP** intermediate prior to the templating step (**ZIP**), so this method requires specific conditions in terms of concentration, solvent, temperature, and reagents in order to prevent off-template reactions. Covalent template-directed synthesis addresses these limitations by connecting the template and substrates with kinetically inert bonds to obtain a more robust pre-**ZIP** intermediate. After the **ZIP** reaction, the covalent linkage holding the template and product together must be cleaved in order to release the product. Covalent templating, therefore, requires high-yielding chemistry to attach the substrates to the template, orthogonal chemistry for the subsequent **ZIP** reaction, and high-yielding chemistry to cleave the product from the template.

We highlight the toolbox of reactions that can be used for the design of efficient covalent templating processes and discuss strategies that favor on-template over off-template pathways. The review charts the evolution of covalent templating since the first synthesis of a [2]catenane reported by Schill in the 1960s. As the examples illustrate, covalent template-directed synthesis now represents an established methodology, providing access to complex molecules with widespread applications in molecular recognition, nanotechnology, and materials science. The examples described here highlight opportunities for the design of covalent templating systems far beyond the current scope. Covalent templating offers a high level of control for the stepwise assembly of complicated molecular architectures and represents a powerful synthetic methodology for the preparation of novel structures, such as covalent organic cages and framework materials, including mechanically interlocked covalent organic frameworks.^123,124^
